# Defective macrophage plasticity exacerbates progression of inflammation in patients with rheumatoid arthritis

**DOI:** 10.3389/fimmu.2026.1869466

**Published:** 2026-10-28

**Authors:** Karina Kulakova, Daphni Doulaptsi Teeuwen, Roozbeh Sanaei, Eoghan McCarthy, Laura Durcan, Cathrine Hughes, Colm Kirby, Michael Freeley, Achilleas Floudas

**Affiliations:** 1 School of Biotechnology, Dublin City University, Dublin, Ireland; 2 Life Sciences Institute, Dublin City University, Dublin, Ireland; 3 Department of Rheumatology, Beaumont Hospital, Dublin, Ireland; 4 Royal College of Surgeons in Ireland, Dublin, Ireland; 5 Department of Rheumatology, Naas General Hospital, Kildare, Ireland; 6 Tallaght University Hospital, Dublin, Ireland; 7 Medical Biology and Immunology group, Laboratory of Biology, Medical School, University of Ioannina, Ioannina, Greece; 8 Biomedical Sciences Institute, FORTH, Ioannina, Greece

**Keywords:** inflammatory arthritis, M2C, macrophage polarisation, plasticity, rheumatoid arthritis

## Abstract

**Background:**

Rheumatoid arthritis (RA) affects over 1% of the global population. Current therapies cannot cure the disease, and only 20% of patients achieve long-term, drug-free remission. Recent studies highlight a dual role for synovial macrophages, which comprise both proinflammatory and remission-associated subsets. However, how macrophage plasticity and metabolic programming drive RA pathogenesis remains poorly understood. This study investigated how phenotypic and functional shifts in macrophage subsets contribute to chronic joint inflammation and bone erosion.

**Methods:**

Peripheral blood CD14+ monocytes from RA patients and healthy controls (HC) were differentiated into M1, M2a, M2b, M2c, and M2d macrophage subsets. To simulate the inflamed joint, cells were cultured under both atmospheric oxygen and hypoxic conditions. Phenotypic and chemokine receptor profiles were evaluated using RT-qPCR and multiparametric flow cytometry for a panel of markers, including CD14, CD11c, CD38, CD86, CD163, CD206, CD209, MerTK, IRF4, CCR1, CCR2, CCR4, CCR5, and CCR8. Metabolic characterization was performed via flow cytometric assessment of protein synthesis. To validate these phenotypes *in vivo*, scRNA-seq analysis (GSE200815) was conducted on synovial tissue macrophages from RA and HC individuals. Finally, the functional capacity of each macrophage subtype to differentiate into bone-resorbing osteoclasts was assessed.

**Results:**

scRNA-seq data identified M1, M2a, M2b, M2c, and M2d clusters at comparable frequencies between HC and RA synovial tissues. However, RA macrophage subsets exhibited a distinct proinflammatory shift, characterized by increased CD86 and VEGF expression, and reduced MerTK expression. *In vitro*, RA-derived macrophages displayed altered metabolic activity and a similar pathogenic phenotype, which was particularly pronounced in the M2c subset compared to HCs. Hypoxia significantly exacerbated these phenotypic discrepancies in RA M2c macrophages. Functional assays revealed that all macrophage subsets, except M2c, successfully differentiated into osteoclasts, with RA-derived cells yielding significantly higher numbers of pathogenic osteoclasts.

**Conclusion:**

Dysregulated phenotypic, metabolic, and functional plasticity causes RA macrophages to adopt intermediate, pathogenic phenotypes. These alterations may impair tissue resolution, enhance osteoclastogenesis, and sustain chronic inflammation in the arthritic joint.

## Introduction

Rheumatoid Arthritis (RA) is a chronic inflammatory autoimmune disease, typically involving progressive inflammation of the joints and significant comorbidities affecting 1% of the global population and can lead to loss of function of the joint if left untreated (1–3). The driving force of RA is the complex interplay between innate and adaptive immune cells alongside dysregulated joint stromal tissue, which combine to produce chronic inflammation and articular destruction (4). This results in excessive autoimmune responses disrupting bone metabolism, which prevents healthy bone formation and favours bone resorption (5). There are no cures for RA with current therapies aiming to alleviate symptoms and improve patients’ quality of life; however, only 20% of patients achieve drug-free remission for over 2 years (1).

Macrophages play a vital role in the innate immune system’s inflammatory response and in maintaining tissue homeostasis. In addition to this, they are a source of proinflammatory cytokines and chemokines activators of other immune cells and secretors of tissue-degrading enzymes, leading to chronic inflammation and pathogenesis of RA (1, 6). Polarisation is a dynamic process where macrophages can take on various functional phenotypes, shifting from proinflammatory (M1) to anti-inflammatory (M2), in response to microenvironmental signals such as cytokines, chemokines and fibroblast-like synoviocytes (FLSs) (7).

In RA, there is an imbalance in the M1/M2 macrophage phenotype and cytokine activity favouring M1 macrophages, leading to the disruption of normal homeostasis between osteoclasts and osteoblasts, in turn causing loss of bone homeostasis and remodelling (8–10). As a result, the number of macrophages in the synovium correlates with disease activity indicators, such as C-reactive protein (CRP), erythrocyte sedimentation rate (ESR) and joint swelling count, whereby patients with RA with M1/M2 ratio >1 have higher CRP and ESR than patients with RA with M1/M2 ratios ≤ 1 and as a result correlate with a worse clinical prognosis (11). However, it is difficult to predict disease progression and treatment outcome based on clinical parameters alone, including who will develop severe, erosive disease or who will respond to treatment.

M1 macrophages secrete proinflammatory cytokines and chemokines, such as interleukin 1β (IL-1β), IL-6, IL-12, IL-23, tumour necrosis factor alpha (TNF-α), and produce reactive oxygen species (ROS), which result in joint erosion, tissue damage and degradation (12). Hence, this subset sustains and mediates the inflammatory process in RA (13). M1 macrophages are associated with increased osteoclast (OC) differentiation due to their excessive production of proinflammatory mediators (14). In a previous study, peripheral blood monocytes from patients with RA were stimulated with macrophage colony-stimulating factor (M-CSF) and receptor activator of NF-κB ligand (RANKL), leading to their differentiation to multinuclear OCs (15). Interestingly, the expression of cytokines such as IL-1β, IL-6 and TNF-α leads to the expression of RANKL, which in turn promotes osteoclastogenesis (16, 17). As a result, M1 macrophages, with the help of OCs, correlate with worse disease progression due to their aggressive effects on joint and cartilage.

On the other hand, several subsets of M2 macrophages have been discovered, namely M2a, M2b, M2c and M2d. They are typically marked by anti-inflammatory mediators such as mannose receptor c-type 1 (MRC1, also known as CD206), cluster of differentiation 163 (CD163), dendritic cell-specific intercellular adhesion molecule-3-grabbing non-integrin (DC-SIGN, also known as CD209) and arginase 1 (Arg1), accentuating their role of resolving inflammation. M2 macrophages can polarise into distinct subtypes in response to differentiation signals driven by stimuli such as IL-4, immune complexes, glucocorticoids/IL-10, and A2 adenosine receptor agonists driving the generation of M2a, M2b, M2c and M2d macrophages, respectively (1, 10, 18–20). M2a macrophages secrete anti-inflammatory cytokines and chemokines, such as IL-10, TGF-β and C-C motif chemokine ligand 17 (CCL17), allowing them to operate in wound healing and allergic reactions (19, 21). They also enhance tissue repair by fibrotic lesion formation and extracellular matrix development via CD206 and fibronectin production (10). M2b macrophages are immunoregulatory cells which promote Th2 differentiation whilst secreting IL-10, IL-12 and CCL1 (19). IL-10 and CCL1 combined allow M2b macrophages to contribute to the development of an anti-inflammatory microenvironment. M2c macrophages exhibit their anti-inflammatory activity by releasing high amounts of IL-10 and transforming growth factor beta (TGF-β), providing M2c’s with regenerative activity for cartilage and bone regeneration (22). They also have high phagocytic capacity for apoptotic cells by upregulating CD163 and macrophage receptor with collagenous structure (MARCO), and overexpression of Mer tyrosine kinase (MerTK) (23, 24). CD163 is a haemoglobin scavenger receptor expressed by monocytic cells that mediates endocytosis of haemoglobin complexes, resulting in a protective detoxification effect with a role in inflammation resolution, as found in high quantities in inflamed tissue (25). Their expression is induced by IL-10 and inhibited by LPS and IFNγ. M2d macrophages secrete anti-inflammatory cytokines, such as IL-10 and TGF-β, providing functions in epithelial healing, whilst also expressing proinflammatory cytokines such as vascular endothelial growth factor (VEGF), thereby playing a role in angiogenesis (26). Communication between M1 and M2d macrophages exists via VEGF and the C-C motif chemokine receptor 1 (CCR1)-CCL3 signalling pathway (27).

Recent evidence highlights novel immunoregulatory functions of macrophages in RA; for instance, MerTK+ macrophages correlate with clinical remission, while CX3CR1+ macrophages form a protective structural barrier in the healthy synovium that becomes disrupted in RA (6, 28). Additionally, Lurier et al. characterised the transcriptomic profile of M2c macrophages relative to M1 and M2a subsets, suggesting a specialized role in early-stage wound healing (23), whereas Feng et al. delineated the distinct involvement of M1, M2a, M2b, and M2c subsets in symptomatic juvenile idiopathic arthritis (JIA) (29). Driven by these insights, this study hypothesises that aberrant macrophage polarisation and plasticity represent critical checkpoints that drive the pathogenic progression of RA. Currently, the intrinsic propensity of peripheral blood-derived monocytes to differentiate into the full spectrum of pro- and anti-inflammatory subsets remains poorly understood. Crucially, no study to date has systematically evaluated the simultaneous induction, of all established macrophage subsets under both atmospheric and microenvironmental hypoxia. Therefore, this study aims to elucidate how macrophage plasticity and hypoxic conditioning collectively drive chronic synovial inflammation in RA.

## Materials and methods

### Patient recruitment

Patients with RA were recruited at Beaumont Hospital (Dublin, IE) and Naas General Hospital (Kildare, IE). Ethical approval was obtained from the Beaumont Hospital Ethics Medical Research Committee and the Naas General Hospital Ethics Committee. Following informed consent, peripheral blood was isolated. A total of 41 patients were enrolled in this study, with their demographics outlined in **Table 1**. The exclusion criteria included patients with other autoimmune diseases and patients currently on Rituximab and/or Abatacept treatment. Healthy control (HC) buffy coats were acquired from the Irish Blood Transfusion Services in St James’s Hospital (Dublin, IE).

**Table 1:** Demographics of the enrolled patients with RA.

Patient Demographics			N	%	-
Total No. of Patients			41	100	–
Male			12	29.27	–
Female			29	70.73	–
			Min	Max	Mean
Age (years)			29	82	63
			**N**	**%**	–
Disease Stage	Active		19	46.34	–
	Low-grade		9	21.95	–
	Remission		13	31.71	–
Serological Status	Positive		35	85.37	–
	Negative		6	14.63	–
Treatment	No treatment		3	7.32	–
	MTX only		13	31.71	–
	MTX combination		10	24.39	–
	Biologics	TNF	6	14.63	–
		IL-6	1	2.44	–
	Metabolic inhibitors		3	7.32	–
	Other		5	12.19	–

A total of 41 patients were enrolled in the study. MTX, methotrexate; TNF, tumour necrosis factor; IL-6, interleukin 6.

### Peripheral blood mononuclear cells (PBMC) and monocyte isolation

Patient blood samples were collected into S-Monovette heparin collection tubes (Sarstedt). The samples were diluted 1:2 and 1:4 with sterile 1x phosphate-buffered saline (PBS) (Corning). PBMC were subsequently isolated from patient’s blood and HC by density gradient centrifugation. Briefly, the samples were under-layered with lymphopure (Biolegend) followed by centrifugation at 700g for 25 minutes at room temperature (RT) with no brake. The interphase layer containing the PBMCs was collected with a sterile Pasteur pipette (Sarstedt), transferred into a new 50ml falcon (CellStar), resuspended with PBS and centrifuged at 650g for 7 minutes at RT with a full brake. Two more washes were performed at 500g and 400g to remove residual platelets, followed by the cell counting and viability assessment of isolated PBMCs with a Neubauer chamber hemacytometer (Knittel Glaeser, Germany).

Subsequently, CD14+ cells were isolated from PBMCs via magnetic CD14 positive selection following the manufacturer’s protocol (MojoSort Human CD14 Selection Kit, Biolegend). Briefly, PBMCs were resuspended in 1x MojoSort Buffer (Biolegend) at a volume of 1x10^8^ cells/ml. CD14+ cells were labelled by incubating the samples with biotin-conjugated anti-human CD14 antibody and streptavidin nanobeads. CD14+ cells were magnetically labelled and retained via magnetic separation from the rest of the cells in the sample. The unlabelled fraction was poured out, and labelled cells were resuspended in MojoSort Buffer before being placed back in the magnet, this step was repeated 3 times. Isolated monocytes were resuspended in complete media (RPMI 1640 with L-glutamine and 25mM HEPES (Corning), 10% fetal bovine serum (FBS) (Merck) and 1% penicillin-streptomycin (Corning)), counted, and seeded into a 24-well flat bottom plate (Greiner) (5x10^5^ cells per well) and a 96-well flat bottom plate (Corning) (1x10^5^ cells per well) for cell culture.

### Macrophage polarisation

CD14+ monocytes were differentiated into proinflammatory macrophages using 50ng/ml human granulocyte-macrophage colony-stimulating factor (GM-CSF) (Biolegend) and anti-inflammatory macrophages using 100ng/ml human M-CSF (Biolegend) and incubated at 37˚C with 5% CO_2_. On day three post isolation the media was changed by centrifugation of the plates at 300g for 5 minutes at RT with a full break, removing the supernatants and adding complete media with 50ng/ml of GM-CSF and 50ng/ml M-CSF to M1 and M2 wells respectively and placed back in the incubator at 37˚C and 5% CO_2_.

Macrophages were polarised on day 5 post isolation into M1, M2a, M2b, M2c and M2d macrophage subsets. M1 macrophages were generated by exposing cells to 20ng/ml human interferon-γ (IFNγ) (Biolegend) and 10ng/ml lipopolysaccharide (LPS) (Merck). M2a macrophages were polarised with 20ng/ml human interleukin-4 (IL-4) (Biolegend). M2b macrophages were polarised with a novel approach utilising immune complexes for citrullinated antibodies. Immune complexes were formed by incubating 1ng/ml citrullinated vimentin (Vim) (Cayman) with 10ng/ml anti-citrullinated vimentin antibody (Anti-Cit Ab) (Abcam) for 1 hour before being added to the M2 macrophage cells. A 10nM solution of corticosteroid, fluticasone propionate (FP) (Merck), was added to macrophages to generate M2c macrophages. M2d macrophages were polarised with 5μM 5’N-ethycarboxyamide adenosine (NECA) (Merck), an adenosine metabolite. Control wells were included for the M2b polarisation by having separate wells polarised with M2 Vim only and M2 Anti-Cit Ab only. The immune complexes’ controls received either 1ng/ml Vim or 10ng/ml Anti-Cit Ab. The cells were incubated for polarising stimulation for 48 hours at 37˚C and 5% CO_2_. Macrophages were harvested on day 7 post isolation.

### Osteoclast differentiation

Following polarisation M1, M2a, M2b, M2c and M2d patient-derived macrophages were differentiated into osteoclasts. All macrophage subsets were exposed to 50ng/ml M-CSF and 100ng/ml RANKL (PeproTech) and incubated at 37˚C and 5% CO_2_. On days 3 and 7 post-osteoclast differentiation, the media was changed via plate centrifugation at 300g for 5 minutes at RT with a full break, removing the supernatants, adding complete media with 50ng/ml M-CSF and 100ng/ml RANKL and incubating at 37˚C and 5% CO_2_. The generated OCs were imaged or lysed on day 10 post-differentiation for future reverse transcription-quantitative polymerase chain reaction (RT-qPCR) analysis.

### Tartrate-Resistant Acid Phosphatase (TRAP) staining

According to the manufacturer’s protocol, the Leukocyte Acid Phosphatase (TRAP) Kit (Sigma) was used to qualitatively analyse and histologically visualise the expression of TRAP by macrophage-derived osteoclasts. After OC differentiation, cell supernatants were removed, and cells were fixed in the 24-well plate with 4% paraformaldehyde (PFA) (Biolegend). Cells were washed, TRAP staining solution was added to the cells and incubated at 37˚C for 1 hour in the dark. Subsequently, counterstaining was carried out using haematoxylin solution for 2 minutes at RT. Examination was performed using a light microscope (Nikon) at 200x and 400x magnification and images were recorded. Cells that stained purple-red were considered TRAP+ OCs and multinucleated cells had ≥ 3 nuclei.

### Reverse Transcription-quantitative Polymerase Chain Reaction (RT-qPCR)

RT-qPCR was performed to investigate the phenotype of the polarised macrophage subtypes. The EasyPure RNA Kit (TransgenBiotech) was used according to the manufacturer’s protocol to isolate RNA from the polarised macrophage and osteoclast cell lysates. The concentration and purity of isolated RNA were quantified by Nanodrop One spectrophotometry (Thermo Fisher Scientific). Following the manufacturer’s protocol, 300ng of extracted RNA was reverse transcribed into cDNA with the EasyScript First-Strand cDNA Synthesis SuperMix kit (TransgenBiotech) using the MiniAmpPlus Thermal Cycler (Applied Biosystems). Gene expression was measured by ChamQ Universal SYBR qPCR Master Mix (Vazyme Biotech) on the LightCycler 96 (Roche Diagnostics) using 15ng of cDNA per sample run for 40 cycles. The RT-qPCR primer panel was designed using primer design for macrophage markers including CD206, IL-10R, Arg1, CD163, VEGF and osteoclast markers such as transcription factor Jun (cJUN), cathepsin K(CTSK), peroxisome proliferator-activated receptor gamma coactivator 1-beta (PGC1β) and nuclear factor of activated T cells 1 (NFATc1) and ordered from Merck. Gene expression was normalised on the glyceraldehyde 3-phosphate dehydrogenase (GAPDH) housekeeping gene values obtained in parallel. PCR primer sequences can be found in **Supplementary Table S1**.

### Flow-based ELISA

A flow-based multiplex enzyme-linked immunosorbent assay (ELISA) assay incorporating the sandwich ELISA approach was utilised for functional profiling of the macrophage subtypes. According to the manufacturer’s protocol, the RayPlex Human Inflammation Bead Array Kit (CliniSciences) was used to quantitatively analyse the expression of IL-6, IL-12, IL-17a, IL-1β and TNFα inflammatory factors from polarised macrophage supernatants in a 96-well round-bottom plate (Corning). This assay uses different-sized beads pre-coated with a capture antibody that binds to the PE-conjugated secondary streptavidin antibody. Protein detection takes place via the PE-streptavidin-biotin-antibody complex, whereby the level of PE fluorescence reflects the amount of protein captured on the beads. Samples were transferred to FACS tubes and acquired on the FACSAria I (BD Biosciences) and the intensity of fluorescence of PE is measured. Data was analysed using the FlowJo software version 10.10.0 (BD Biosciences). The cells were gated based on forward and side scatter, followed by the removal of doublets and dead cells. Subsequently, the cell populations were segregated by bead size, and each cytokine was analysed individually. The protein was quantified through the comparison of the PE signal to the standard curve generated from purified protein standards of known concentrations.

### Metabolic analysis

The flow-based metabolic analysis studied single-cell energetic metabolism by utilising inhibition of protein synthesis. The assay is performed *ex vivo* on a 96-well plate. Macrophages were incubated with metabolic inhibitors at concentrations of 100mM 2-Deoxy-D-Glucose (2DG) (Merck) and 1µM Oligomycin A (Oligo) (Merck) for 15 minutes at 37 ˚C and 5% CO_2_. To measure protein synthesis levels 10µg/ml of Puromycin Dihydrochloride (Puromycin) (Merck) was added to the cells and incubated for 30 minutes at 37˚C and 5% CO_2_. Supernatants were removed from cells, washed with PBS and incubated with accutase (Biolegend) for 10 minutes at 37˚C and 5% CO_2_. Subsequently, macrophages were detached from the 96-well flat bottom plate by pipetting and transferred to a new 96-well round bottom plate for extracellular staining. The cells are centrifuged, and supernatants are decanted. PBS is used to wash away any traces of accutase following another centrifugation. The Zombie NIR Fixable viability dye (Biolegend) was diluted 1:300 in PBS, added to the cells, and incubated in the dark for 30 minutes at 4˚C. Cells were washed with PBS, centrifuged and supernatants decanted. Human Truestain FCX (Biolegend) was used to eliminate nonspecific binding to the Fcγ receptor. Based on the manufacturer’s instructions, it was diluted 1:10 in fluorescence-activated cell sorting (FACS) buffer (Biolegend), added to the cells and incubated in the dark for 10 minutes at 4˚C, followed by another wash with PBS. Cells were fixed and permeabilised using the FOXP3 Fix/Perm Buffer set (Biolegend). Cells were first fixed with the FOXP3 Fix/Perm buffer diluted 1:4 with PBS, incubated in the dark for 30 minutes at 4˚C, followed by permeabilisation with the FOXP3 Perm buffer diluted 1:10 with PBS and centrifuged. Anti-Puromycin-PE antibody (Biolegend) was diluted 1:12 with 1x FOXP3 Perm buffer, added to the cells and incubated in the dark for 40 minutes at 4˚C. Macrophages were subsequently washed with 1x FOXP3 Perm buffer twice and once with FACS buffer before being transferred into FACS tubes (Corning).

Cells were acquired on the FACSAria I (BD Biosciences) and analysed using the FlowJo software version 10.10.0 (BD Biosciences). Single-stained controls were used for compensating spectral overlap between fluorochromes. Doublet exclusion was performed to retain single cells. The Zombie NIR Fixable viability dye (Biolegend) was used to gate out the dead cells to decrease non-specific binding and false positive results. Changes in metabolism affect adenosine triphosphate (ATP) levels and hence their capacity for protein synthesis which is calculated based on geometric mean fluorescence intensity (MFI). Calculations were performed as described previously (30). Glucose dependence was calculated using the following equation: Glucose dependence (%) = 100*((dimethyl sulfoxide control (DMSO Co) – 2DG)/(DMSO Co – 2DG+O)). Mitochondrial dependence was calculated with the following equation: Mitochondrial dependence (%) = 100*((DMSO Co – Oligo)/(DMSO Co – 2DG+O)). Glycolytic capacity was calculated as follows: Glycolytic capacity (%) = 100 – (100*((DMSO Co – Oligo)/(DMSO Co – 2DG+O)). Fatty acid oxidation (FAO) and amino acid oxidation (AAO)capacity were established with this equation: FAO&AAO capacity (%) = (100-(100*((DMSO Co – 2DG)/(DMSO Co – 2DG+O)).

### Flow cytometric analysis

Macrophages polarised into M1, M2a, M2b, M2c and M2d were stained using a panel of fluorochrome-conjugated antibodies against extracellular and intracellular markers for flow cytometric analysis. The following antibodies were used: aCD14, aCD11c, aCD38, aCD86, aCD163, aCD206, aCD209, aMerTK, aIRF4, aCCR1, aCCR2, aCCR4, aCCR5 and aCCR8, as demonstrated in **Supplementary Table S2** (Biolegend and BD Biosciences).

Supernatants were removed from cells, washed with PBS, then detached from the 96-well flat bottom plate with accutase (Biolegend) and incubated for 10 minutes at 37˚C at 5% CO_2_. Subsequently, macrophages were transferred to a new 96-well round bottom plate for extracellular staining. Cells were centrifuged and resuspended with PBS. The Zombie NIR Fixable viability dye (Biolegend) was added to the cells as per the manufacturer’s instructions, and incubated in the dark for 30 minutes at 4˚C. Human Truestain FCX (Biolegend) was added to the cells and as per manufacturer’s instructions, incubated in the dark for 10 minutes at 4˚C. Fluorochrome-conjugated antibodies targeting surface markers were added to the macrophages and incubated in the dark for 30 minutes at 4˚C. Cells were subsequently washed with FACS buffer twice. If intracellular staining was required, the cells were fixed and permeabilised using the intracellular FOXP3 Fix/Perm Buffer set (Biolegend) as per the manufacturer’s instructions. Following incubation with the FOXP3 Fix/Perm buffer for 30 minutes at 4˚C, the cells were washed with FOXP3 Perm buffer. Subsequently, antibodies for intracellular staining were added to the cells for 30 minutes at 4˚C. Cells were once again washed with FOXP3 Perm buffer and afterwards once with FACS buffer. In the case that only extracellular staining was required, cells were fixed using 1% PFA fixation buffer (Biolegend) and incubated in the dark for 10 minutes at 4˚C. Samples were then washed in FACS and centrifuged at 300g with the supernatants decanted. Cells were resuspended in FACS buffer and transferred to FACS tubes (BD Biosciences). Samples were acquired using the CytoFlex LX (Beckman Coulter Life Sciences) and analysed using the FlowJo software version 10.10.0 (BD Biosciences).

Gating was performed based on Fluorescence Minus One (FMO) controls and unstained live cells where FMOs were unavailable. Single-stained controls were made using beads (BD Biosciences) and live cells for the live/dead and were subsequently used to adjust spectral overlap between detectors during compensation. Macrophages were gated based on forward and side scatter followed by the removal of doublets and dead cells. Boolean gating was performed for the panel of chemokine receptors. CSV files were generated and utilised for analysis and the generation of simplified presentation of incredibly complex evaluations (SPICE) plots (31).

### Hypoxia

Macrophage polarisation, as described above, was performed under hypoxic conditions in the Baker Ruskinn Invivo2–400 hypoxia chamber (Baker Co) set to 2% O_2,_ 5% CO_2_ and 37˚C to simulate the hypoxic inflammatory environment in the synovial tissue of patients with RA (32). Polarisation under hypoxia was matched with parallel polarisation of cells under atmospheric conditions of O_2_.

### Single cell RNA-seq analysis of publicly available data

RNA-seq analysis was performed in RStudio (version 2024.12.0) to compare the expression of M1, M2a, M2b, M2c, and M2d markers of synovial tissue macrophages of patients with RA and HC from synovial biopsies from knee arthroscopy. The scRNAseq analysis utilised a publicly available scRNAseq dataset of HC and patients with RA from NIH GEO datasets (GSE200815) (7, 33). Each Seurat object representing an individual patient and HC underwent filtering by removing genes detected in less than 3 cells using the EmptyDrops function (version 1.12.1). The samples underwent doublet exclusion using DoubletFinder (version 2.0.3), which compares transcriptional data with a set of artificial doublets, created through the averaging of gene expression profiles of randomly paired cells, to detect and identify true cell doublets (34). Afterwards, dead cell exclusion with mitochondrial associated gene expression cut off at 15% was carried out. The Seurat objects were scaled and normalised with SCtransform (version 0.4.1), resulting in a cohort of 64,048 cells for downstream analysis. Sctransform uses non-heuristic approaches for data normalisation based on Pearson residuals from a negative binomial regression while regressing out cell-specific confounding biological covariates, such as mitochondrial read percentages (35). The individual patient and HC samples were merged into a single Seurat object. Principal component analysis (PCA) dimensionality reduction was performed on the scaled Seurat object to simplify the complex dataset into fewer components that retain the most significant variance, thereby facilitating visualisation and enabling robust clustering. Integration was performed with RPCAIntegration in Seurat (version 5.1.0). Reciprocal PCA (RPCA) is highly efficient and resilient in overcoming over-correction by projecting each batch into PCA spaces to identify true shared biological “anchors” whilst retaining cell frequency variation. The synovial cell clusters were identified with FindClusters in Seurat and visualised on a UMAP plot. The cell clusters were analysed for expression of known markers associated with the immune cells and manually annotated based on differentially expressed gene (DEG) lists derived using the FindAllMarkers function of Seurat with the Wilcox test and p-values adjusted by Bonferroni correction. DEGs were filtered with the following criteria: minimum 0.25 cell cluster expression, minimum log2 fold change of 0.25 and a p value below 0.05.

Subsequently, the macrophage cluster was subsetted from the rest of the synovial cell clusters for downstream analysis. The Seurat object was re-normalised, re-scaled and re-integrated with Harmony (version 1.2.3) to allow for further re-clustering of the macrophage cells. Harmony carries out cell-specific correction of the cell’s PCA coordinates, which thereby reduces variation associated with technical differences between samples and applies linear correction based on the sample of origin (36). This effectively mixes individual donors within the shared clusters without masking underlying physiological responses or disease states. The macrophage cluster was re-clustered and visualised on a UMAP plot. DEG profiles was generated for each cluster using the FindAllMarkers function. Macrophage subsets were annotated based on DEG expression and prior knowledge of M1, M2a, M2b, M2c and M2d macrophage marker expression. Macrophage subset distribution was compared between RA and HC based on the number of cells per cluster and visualised using ggplot2 (version 3.5.1). Cluster analysis was performed by comparing the expression of macrophage markers associated with RA in the macrophage subclusters and visualised in Violin plots.

The distinct macrophage subclusters were isolated into individual Seurat objects. Pathway enrichment analysis was performed using pathfinder (version 2.4.1) on each macrophage subtype (37). A DEG profile was derived for each cluster, comparing the differentially expressed genes in patients with RA to HCs. The resolution of DEGs were filtered based on log2 fold change and adjusted p value followed by run_pathfindR based on the KEGG database. The enriched pathways were visualised using the enrichment_chart plots.

### Statistical analysis

Statistical analysis was performed using the GraphPad Prism 10 software version 10.5.0 (GraphPad). Data was expressed as the mean ± SEM of at least three independent experiments for phenotypic, metabolic and functional analysis. Two-way ANOVA and paired Student’s t-test were utilised for statistical significance evaluation. P values of less than 0.05 were considered statistically significant.

## Results

### Defective macrophage polarisation in patients with RA compared to HC.

CD14+ monocytes were isolated from patients with RA and HC blood, differentiated into macrophages, and polarised into all known macrophage subtypes following the protocol depicted in **Figure 1A**. Differential transcriptomic profiles can be observed between HC and patients with RA M1 and M2 subtype macrophages (**Figure 1B**). Based on the literature, it is expected to observe high VEGF expression in M1, high CD206 expression in M2a, high IL-10R levels in M2b, high CD163 and Arg1 in M2c and high VEGF in M2d macrophages (19, 20, 26, 38–42). M1 macrophages are expected to have low expression of M2-related markers such as CD163 and Arg1 (6, 41). Meanwhile, low expression of VEGF is expected in M2a, M2b and M2c macrophages (20, 26, 39).

**Figure 1:**
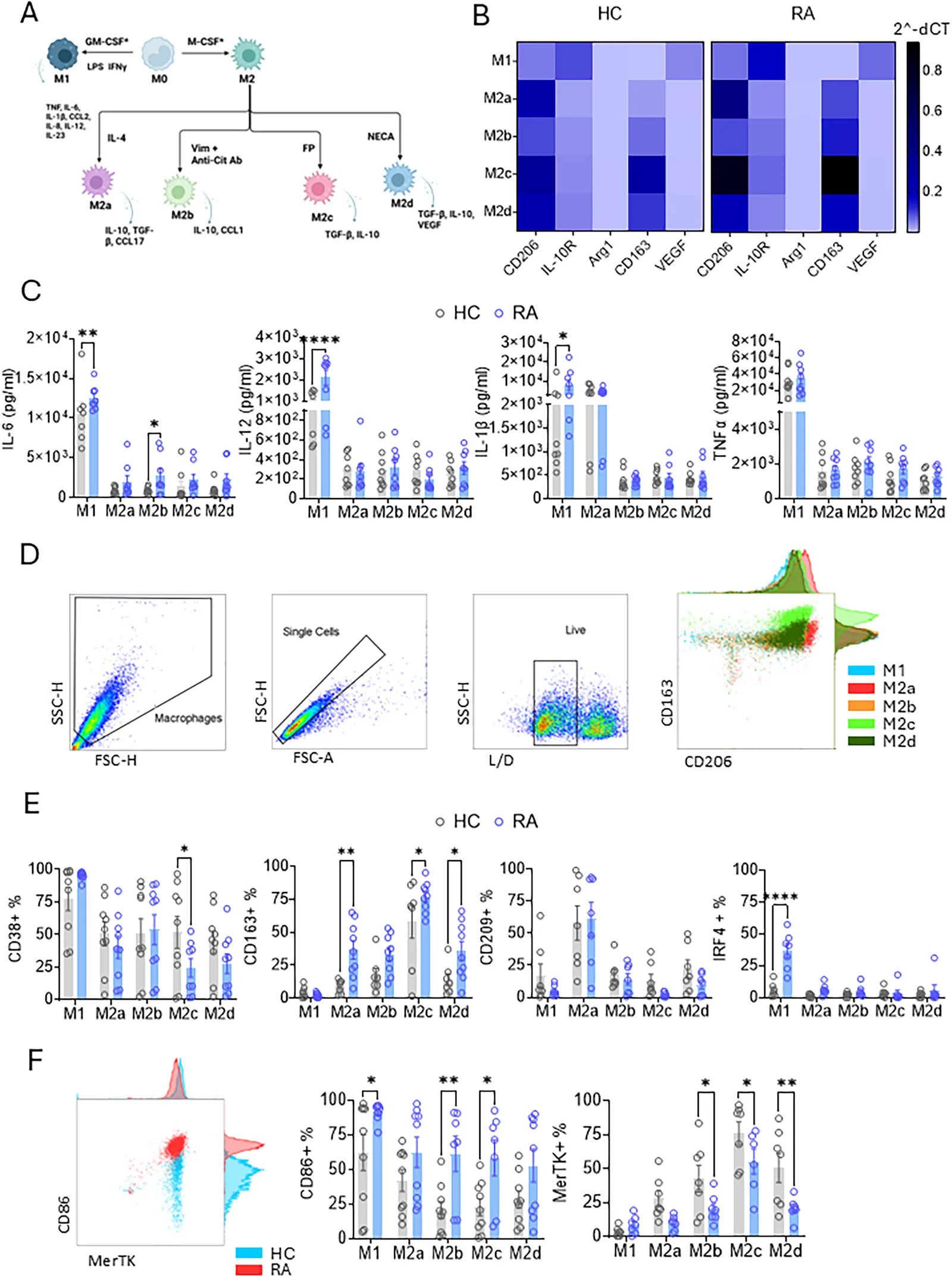
Differential macrophage plasticity in patients with RA compared to HC. **(A)** Schematic of macrophage differentiation and polarisation. PBMCs were isolated from patients with RA and HC blood, and monocytes were subsequently isolated. Monocytes were differentiated into M1 and M2 macrophages using granulocyte-macrophage colony-stimulating factor (GM-CSF) and macrophage colony-stimulating factor (M-CSF), respectively. Macrophage differentiation was employed for 5 days from the initial monocyte isolation, followed by 2 days of macrophage polarisation into M1 [lipopolysaccharide (LPS) and interferon-gamma (IFN-y)], M2a [interleukin 4 (IL-4)], M2b [citrullinated vimentin (Vim) and anti-citrullinated vimentin antibody (Anti-Cit Ab)], M2c [fluticasone propionate (FP)] and M2d [5’N-ethycarboxyamide adenosine (NECA)]. **(B)** Heatmap of relative gene expression assayed via RT-qPCR and displayed as 2^-dCT values of HC (n=8) and RA (n=9) macrophages, ranging from 0 to 1, representing low and high marker expression, respectively. **(C)** Bar charts of flow cytometric MFI in HC (n=8) and patients with RA (n=8) analysing inflammatory cytokine (IL-6, IL-12, IL-1B and TNFα) expression of polarised macrophage subsets. **(D)** Representative flow cytometric analysis gating strategy for the identification of live single-cell macrophages and CD163+CD206+ macrophages. **(E)** Bar charts of flow cytometric percentage of positive cells for markers associated with RA (CD38, CD163, CD209 and IRF4) in HC (n=9) and RA (n=9) macrophages. **(F)** Co-expression of CD86 and MerTK in M2c macrophages for patient with RA and HC macrophages with an associated bar chart of the percentage positive cells for CD86 and MerTK. Statistical analysis was performed by using 2-way ANOVA, *p < 0.05, **p < 0.01, ****p < 0.0001.

HC M1 macrophages displayed high expression of VEGF (M2a *p = 0.0147, M2b **p = 0.0079, M2c *p = 0.0354) and lower expression of CD163, anti-inflammatory cytokines, compared to the M2 subtype macrophages (**Supplementary Figure 1A**). CD206 is highly expressed in M2a (*p = 0.0443) and M2c (*p = 0.02) macrophages, with significantly higher expression compared to M2b subtype macrophages (**Supplementary Figure 1A**). Additionally, M2c macrophages displayed the highest expression of CD163 compared to all other macrophage subtypes. HC M2b and M2d showed similarly high expression of IL-10R and CD163. Additionally, M2d macrophages had higher CD163 expression compared to M1 (*p = 0.048) and M2a (*p = 0.0427) macrophages (**Supplementary Figure 1A**). On the other hand, the expression profile of the RA cohort shows evident variation from the HC group (**Figure 1B**). M1 macrophages in RA show comparable expression of markers to the HC, whereas the M2 subsets are dissimilar. The expression of CD206 and CD163 was further upregulated in patients with RA M2a and M2c macrophages, with a significant increase in CD163 expression by M2c RA macrophages compared to HC (*p = 0.0417) (**Figure 1B**; **Supplementary Figure 1B**). A tendency for higher IL-10R expression was seen in patient-derived M2b compared to their HC-derived macrophage counterparts, respectively (**Figure 1B**). Additionally, a significant increase in IL-10R (*p = 0.0315) and VEGF (*p = 0.0202) expression was observed in RA M1 macrophages compared to HCs, whilst comparable expression was observed in all M2 subtypes (**Supplementary Figures 1A, B**). All M2 subsets demonstrated higher CD206 and CD163 expression in patients with RA compared to HC (**Figure 1B**; **Supplementary Figure 1B**).

To further test the functional differences between patient-derived and HC macrophage subsets, inflammatory cytokine profiling was carried out using a multiplex flow-based ELISA on the supernatants of polarised macrophages. Irrespective of source, M1 macrophages showed significantly higher expression of all proinflammatory cytokines compared to their corresponding M2 subsets (**Figure 1C**). Additionally, patient-derived M1 macrophages had significantly higher expression of IL-6 (**p = 0.0055), IL-12 (****p = 0.0001) and IL-1β (*p = 0.0115) in comparison to the HC derived ones (**Figure 1C**). M2 macrophages in both groups have decreased cytokine expression in comparison to M1 macrophages; however, variation is seen between the different subsets. IL-6 is directly linked to RA progression, with levels correlating to joint destruction and disease activity (43). A significant upregulation of IL-6 was observed in M2b (*p = 0.0419) RA-derived macrophages, whilst the other M2 subsets had no significant change in expression (**Figure 1C**). IL-12, commonly produced by macrophages responsible for NK and T cell activation and driving cell-mediated immunity (44), showed comparable expression between patient and HC-derived M2 macrophages. IL-1β is strongly associated with driving joint damage in RA and enhancing the M1 proinflammatory phenotype. Interestingly, M2a macrophages expressed similarly high levels of IL-1β as HC M1 macrophages, whilst the other subtypes demonstrated low cytokine expression (**Figure 1C**). TNFα, IL-1β and IL-6 are known to be involved in mediating M1 macrophage polarisation via the TLR-4-induced nuclear factor kappa-B (NF-κB) signalling pathway (9).

Most macrophages, irrespective of their phenotype, were CD163+CD206+ as highlighted by flow cytometric analysis (**Figure 1D**). Small shifts are seen in CD163 and CD206 expression, with the highest CD163 and CD206 expression seen in M2c and M2a macrophages, respectively (**Figure 1D**). CD38 is an M1 macrophage marker involved in RA pathogenesis by enhancing inflammatory cytokine secretion and contributing to a pathogenic feedback loop by increasing Th17 cells’ pathogenicity via TNFα release (45). Subtype-specific decrease in CD38 expression was observed in RA M2c (*p = 0.0443) macrophages compared to HC (**Figure 1E**). In line with the gene expression analysis (**Figure 1B**; **Supplementary Figure 1B**), significant upregulation of CD163 expression was observed in RA-derived M2a (**p = 0.0013), M2c (*p = 0.0265) and M2d (*p = 0.0118) macrophages compared to their HC counterparts (**Figure 1E**). CD209 surface marker is typically found on M2 macrophages and is induced by IL-4 (46, 47). It has a dual role in RA as its expression correlates with the severity of cartilage damage and can modulate the anti-inflammatory effects of tissue repair in an environment dominated by M2 macrophages (46, 47). The highest expression of CD209 was seen in M2a HC (M1 **p = 0.0026, M2b **p = 0.0028, M2c ***p = 0.0007, M2d *p = 0.0106) and RA (M1 ****p = 0.0001, M2b ***p = 0.0004, M2c ****p = 0.0001, M2d ****p = 0.0001) macrophages compared to the other subtypes; however, no statistical differences were observed between RA and HC macrophages. (**Figure 1E**). IRF4 transcription factor acts as an anti-inflammatory regulator by suppressing M1 polarisation (48). However, a significant upregulation of IRF4 in M1 (****p = 0.0001) patient-derived RA macrophages compared to HCs was observed (**Figure 1E**). CD86 co-stimulatory molecule engages CD28, which is essential for T cell activation (49, 50). Previous studies have shown increased CD86 expression in patient with RA M1 macrophages (39). A significant increase in CD86 expression by most, but not all RA macrophage subsets was observed, specifically M1 (*p = 0.0264), M2b (**p = 0.0045) and M2c (*p = 0.0119) macrophages compared to HC (**Figure 1F**). Furthermore, MerTK plays a role in anti-inflammatory signalling by suppressing NF-κB-driven cytokine production and pro-resolving signalling via the ERK-dependent pathway (51). It also drives efferocytosis by recognising apoptotic cells via bridging Gas6 and Protein S, which bind phosphatidylserine on apoptotic cells (40). Importantly, MerTK was found to be significantly decreased by M2b (*p = 0.0193), M2c (*p = 0.0345) and M2d (**p = 0.0019) RA macrophages in comparison to HCs (**Figure 1F**).

### Chemokine receptor expression by patient with RA and HC macrophage subsets.

Chemokine receptors are vital for macrophage function as they orchestrate their recruitment and migration to the site of inflammation, thereby playing an important role in RA pathogenesis. CCR1, a chemoattractant receptor with a role in the bone microenvironment via its ligand CCL3, had elevated expression in M2c (**p = 0.0074) and M2d (*p = 0.0248) RA-derived macrophages compared to HC, whilst M1 macrophages showed the lowest expression (**Figure 2**). CCR2, critical for macrophage migration, showed reduced expression on the surface of RA M2a (****p = 0.0001) and M2d (*p = 0.0129) macrophages compared to HC (**Figure 2**). A significant increase in CCR4 expression was observed in M1 (****p = 0.0001) RA macrophages compared to HC, whilst the M2 subsets showed no variable differences between HC and patients with RA (**Figure 2**). Patients with RA showed significantly reduced expression of CCR5 in M1 (**p = 0.0022) and all M2 subsets (M2a *p = 0.034, M2b *p = 0.0122, M2c *p = 0.0171, M2d *p = 0.0181) compared to HC (**Figure 2**). No significant variation in CCR8 expression was observed between HC and patients with RA-derived macrophages.

**Figure 2:**
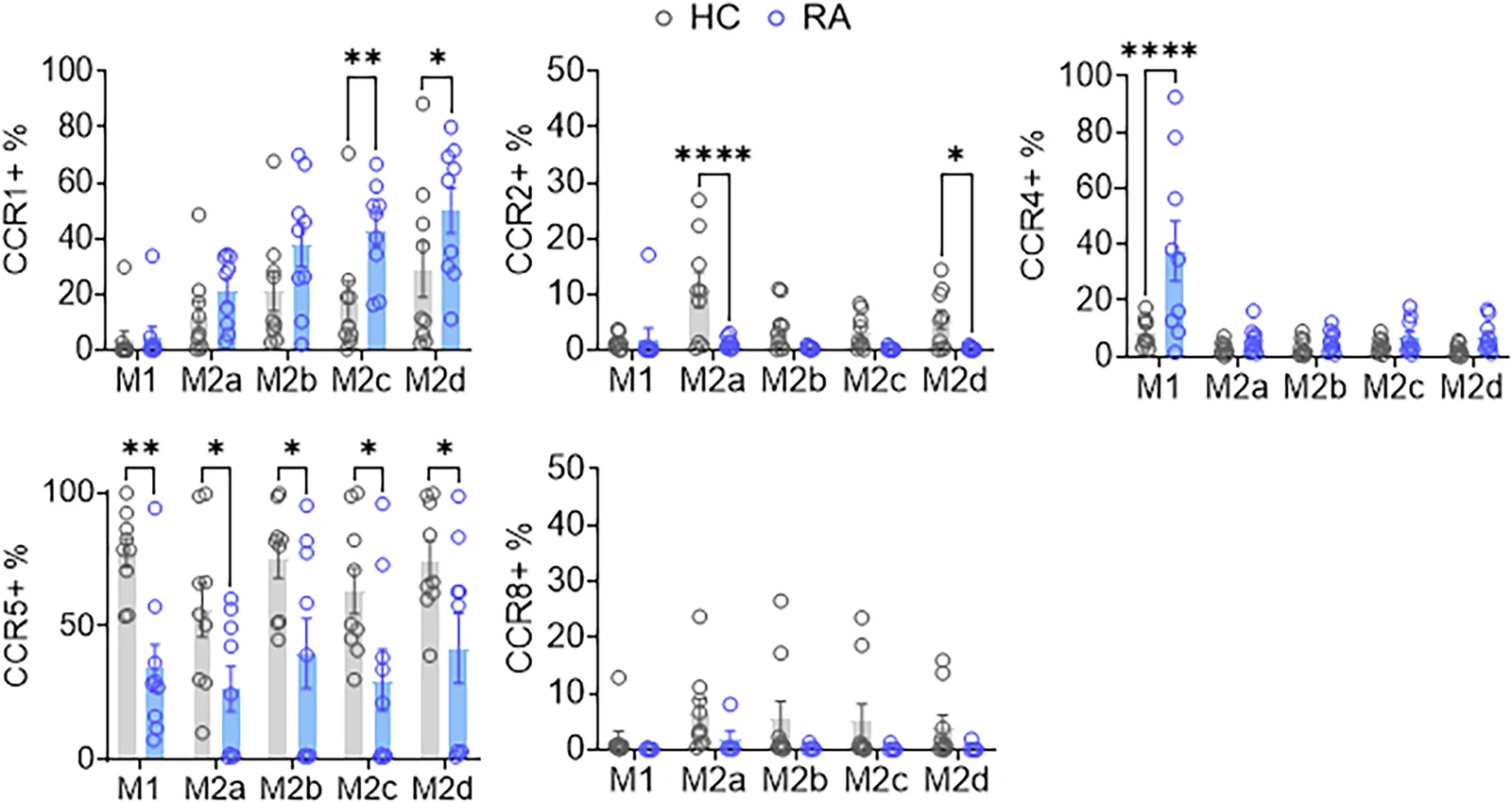
Differential pattern of chemokine receptor expression in patients with RA macrophages compared to HC. Bart charts of flow cytometric percentage of positive cells expressing macrophage-associated chemokines (CCR1, CCR2, CCR4, CCR5 and CCR8) in HC (n=9) and RA (n=9). Statistical analysis was performed by using 2-way ANOVA, *p < 0.05, **p < 0.01, ****p < 0.0001.

Chemokine expression patterns were additionally analysed and presented by SPICE plots (**Supplementary Figure 2**). The pie segments represent the percentage of macrophages that express the corresponding chemokine receptors. The pie arch signifies the chemokine receptors being expressed, and overlaps in the arches are cells that express multiple receptors simultaneously. Differences in chemokine expression patterns are seen in macrophage subsets between RA and HC, with a significant decrease in CCR5 expression by all RA macrophages (**Figure 2**). In addition to this, there is a significant increase in the proportion of macrophages negative for all chemokine receptors tested in M1 and M2a patient-derived macrophages compared to HC (**Supplementary Figure 2**).

### Shifts in metabolism in patient with RA macrophages.

To test the relationship between metabolism and macrophage polarisation, macrophages were treated with inhibitors of glycolysis or oxidative phosphorylation (OxPhos) and changes in protein synthesis were assessed (**Figure 3A**). 2DG is an analogue of glucose which when incorporated cannot be catabolised and blocks glycolysis whereas Oligomycin blocks OxPhos. Puromycin enters the ribosome instead of tRNA and incorporates itself into the synthesising protein upon which it blocks all subsequent protein synthesis in the cell, thereby allowing for the detection of actively translating ribosomes into mRNA via direct immunofluorescence. Cell populations that are energetically dependent on a certain metabolic pathway demonstrate drops in ATP concentration and subsequently protein synthesis levels when the corresponding pathway is inhibited (**Figure 3B**). A leftward shift in puromycin MFI indicates a decrease in protein synthesis in response to the metabolic treatment and hence highlights that the cell population heavily relies on that specific metabolic pathway as a source of energy. Cells treated with DMSO act as a control where protein synthesis runs at maximum capacity. On the other hand, shifts in puromycin MFI are seen for cells treated with 2DG, Oligo and 2DG+Oligo, thereby highlighting variation in the cells’ dependence on glycolysis and OxPhos (**Figure 3B**). The 2DG+Oligo treatment simultaneously targets glycolysis and mitochondrial respiration, causing the cells to starve of ATP and completely stopping protein synthesis highlighted by the major shift in MFI. No puromycin cells show the greatest shift in puromycin MFI due to never being exposed to puromycin and hence act as a negative control. Hence, this assay allows for the measurement of glucose dependency, mitochondrial dependency, glycolytic capacity and FAO (30).

**Figure 3:**
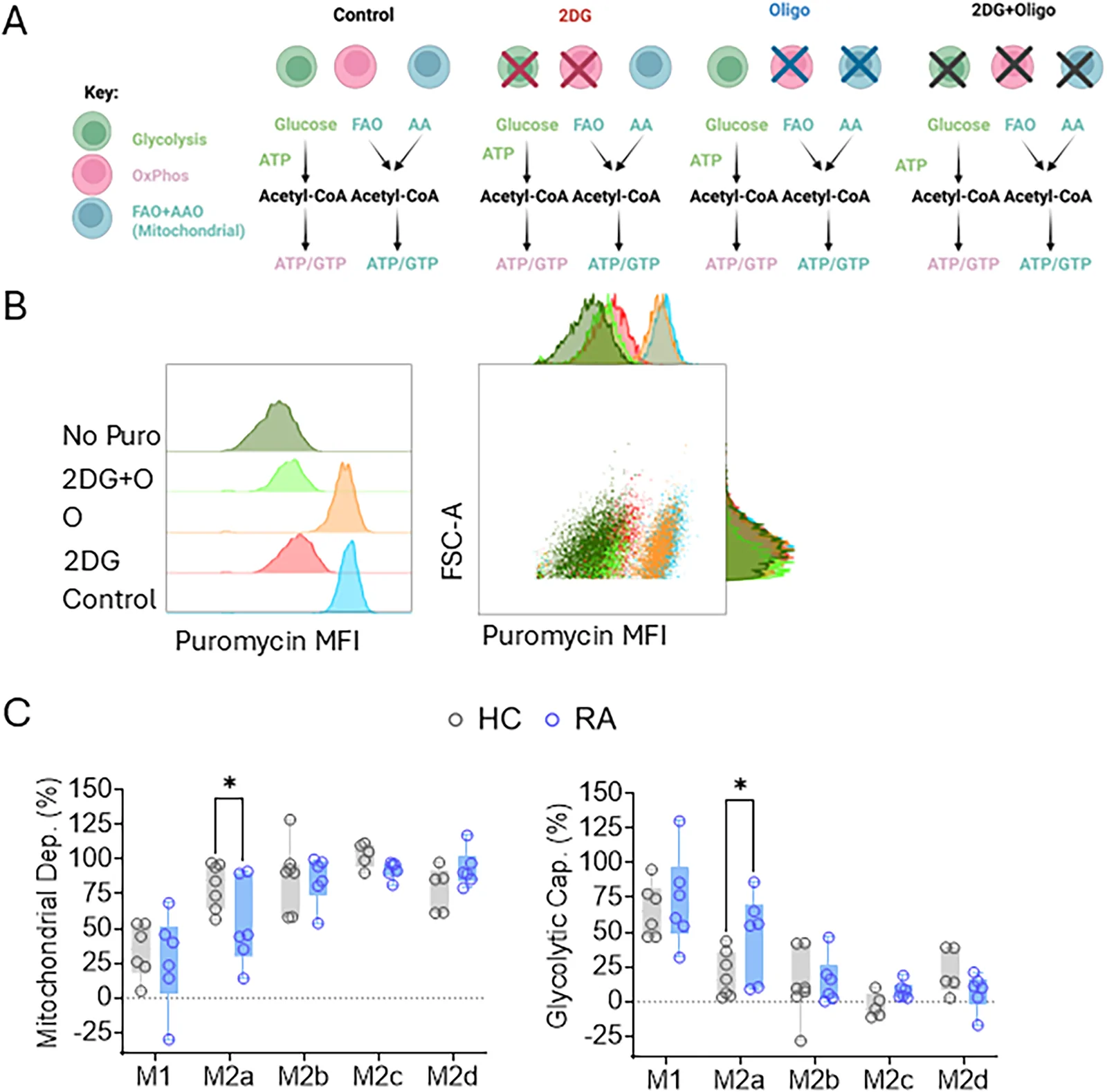
Metabolic analysis of patients with RA vs HC demonstrates plasticity of macrophages. **(A)** Schematic of the flow-cytometry based metabolic assay carried out that utilises metabolic inhibitors, 2DG (2-Deoxy-D-Glucose) and Oligo (Oligomycin A), to inhibit ATP production by blocking glycolysis and OxPhos, respectively. Cells dependent on the inhibited pathway have reduced ATP concentration which in turn significantly impairs protein synthesis, as ATP and protein synthesis levels are tightly correlated. When puromycin enters the ribosome, it gets incorporated into the growing protein chain, causing premature termination of protein synthesis. By measuring puromycin levels, protein synthesis in the different treatment conditions can be monitored, and thus, a drop in protein synthesis signifies how much the cells depend on a certain pathway. **(B)** Flow cytometric plots demonstrate the effects of different assay conditions (Control (DMSO), 2DG+O (Oligo), O (Oligo), 2DG, No Puro (Staining Control)) on macrophage protein synthesis based on puromycin incorporation in the ribosome. Based on the MFI of puromycin, glucose dependence, FAO/AAO, mitochondrial dependence and glycolytic capacity can be calculated. **(C)** Bar charts highlighting the metabolic profiles of macrophage subsets in HC (n=7) and patients with RA (n=6) in terms of their mitochondrial dependence and glycolytic capacity calculated from the MFI of puromycin in the different treatments, with the biggest variation between the two conditions seen in M2a macrophages. Patients with RA M2a macrophages have lower mitochondrial dependency and higher glycolytic capacity, indicating that they are relying more on glycolysis than their HC counterparts. Statistical analysis was performed by using 2-way ANOVA, *p < 0.05. ATP, adenosine triphosphate; OxPhos, oxidative phosphorylation; DMSO, dimethyl sulfoxide; Puro, puromycin; MFI, mean fluorescence intensity; FAO, fatty acid oxidation; AAO, amino acid oxidation.

In HC M1 macrophages demonstrate higher glycolytic capacity and lower mitochondrial dependency compared to M2 macrophages (**Figure 3C**). HC M2 macrophage subsets display similar metabolic profiles to one another, which include high mitochondrial dependency and low glycolytic capacity (**Figure 3C**). No significant deviation was observed in M1, M2b and M2c RA-derived macrophages compared to HC. In fact, M1 macrophages maintained their utilisation of glycolysis and M2b and M2c macrophages depended on mitochondrial metabolism in patients with RA. Interestingly, patient with RA M2a macrophages have an intermediate metabolic profile between an M1 macrophage and a HC M2a macrophage, whereby they had higher glycolytic capacity (*p = 0.0203) and lower mitochondrial dependency (*p = 0.0203) compared to HCs and other M2 macrophages. The M2d subset, on the contrary, showed a tendency for higher mitochondrial dependency and lower glycolytic capacity than M2a, M2b and M2c macrophages. M2b and M2c macrophages demonstrated similarly low levels of glycolytic capacity and high mitochondrial dependency to one another.

### Macrophage subsets are represented in the synovial tissue.

We analysed publicly available scRNAseq data of patients with RA (n=4) and HC (n=5) synovial tissue biopsies (7, 33). The macrophage cluster was identified by the significant expression of macrophage-associated genes (CD86, IL-10RA, IL-1β, CD206, CD163, MerTK) (**Figures 4A, B**). Macrophages were subsequently isolated, normalised, scaled, integrated and re-clustered. M1, M2a, M2b, M2c and M2d macrophage subset clusters were identified based on DEGs (**Figure 4C**). Macrophage cluster annotation was based on the expression of IL-1β, TNFα, IL-6R, NF-κB, VEGFA, TLR2 and TLR4 in the M1 cluster; CD206, CD163, STAT6, S100A6 and MARCO by M2a cells; CD9, S100A6 and SPP1 in M2b subset; CD163, MerTK, CD68, PEGAM1 and CCL18 in the M2c cluster, and VEGFA, CXCR4, SOCS3, EREG in M2d. Surprisingly, macrophage subset distribution was comparable between RA and HC. No significant change in the frequency of the different clusters in HC and RA was observed (**Figure 4D**). Although cluster frequency did not differ, global and cluster-specific transcriptomic differences were observed in RA synovial macrophages compared to HC (**Figure 4E**). CD206 was globally downregulated in all RA macrophages compared to HC macrophage subsets, whilst the highest expression of CD206 remained in M2a macrophages. Global upregulation of CD163 was observed in all except the M2b cluster of patient synovial macrophages compared to HC. RA-derived M2 synovial macrophages showed increased VEGF and CD86 expression, whilst remaining phenotypically distinct from M1 (**Figure 4E**). MerTK expression was high in M2a and M2c macrophage clusters; however, were depleted in patients with RA macrophages (**Figure 4E**). Synovial macrophage expression patterns of CD86 and MerTK are in line with the *in vitro* polarised macrophages (**Figure 1F**).

**Figure 4:**
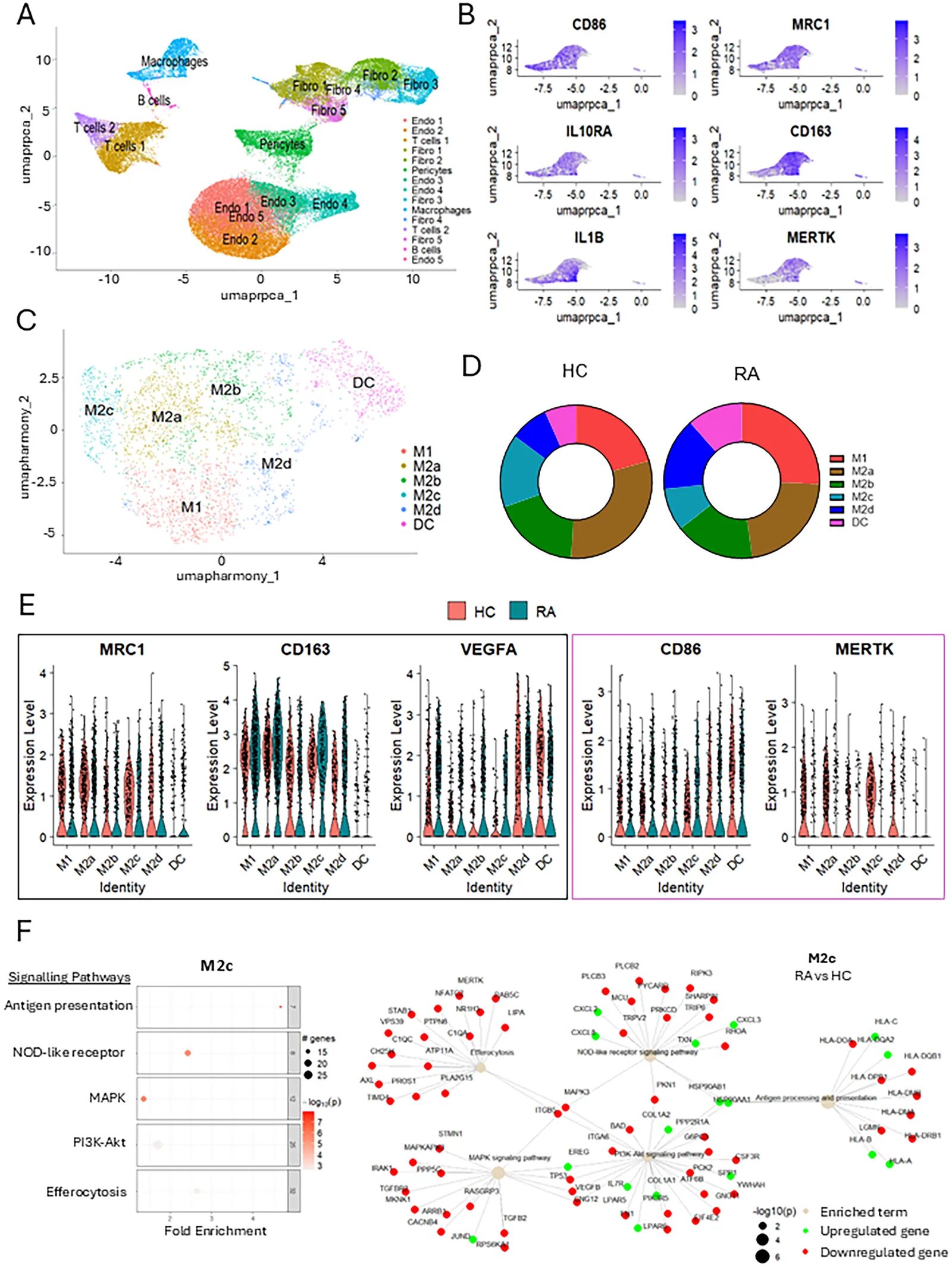
*In vitro* M1 and M2 subsets are matched with macrophage clusters found in synovial tissue. **(A)** Global UMAP of the different cell clusters present in the synovium of HC (n=5) and patients with RA (n=4) from GSE200815. The scRNAseq raw data from each RA patient and HC underwent filtering, doublet exclusion, dead cell exclusion, scaling and normalisation before being merged into a single Seurat object. The object was subjected to dimensionality reduction and integration before the clusters were identified and a UMAP of the cell clusters was generated. Cell clusters were manually annotated based on differentially expressed genes (DEGs) of each cluster. **(B)** Feature plots of macrophage markers (CD86, CD206 (also known as mannose receptor C type 1, MRC1), IL-10RA, CD163, IL-1B and MerTK (Mer tyrosine kinase)) identified to be solely expressed in the macrophage cluster in the global UMAP of cells identified in the synovium. **(C)** The identified macrophage cluster was isolated from the rest of the synovial cell clusters, re-normalised, re-scaled and re-integrated. A new UMAP of macrophage subclusters was generated and labelled based on the DEGs in each cluster. **(D)** Pie chart displaying a comparative distribution of macrophage subclusters in the macrophage Seurat object based on the number of cells per cluster between HCs and patients with RA. **(E)** Violin plots depicting the expression level of DEGs (CD206, CD163, VEGFA, CD86 and MerTK) in macrophage subclusters with a highlight of global upregulation of CD86 and downregulation of MerTK in RA synovial macrophages compared to HCs. **(F)** Individual macrophage subclusters were isolated into separate Seurat objects and were subjected to pathway enrichment analysis comparing DEGs in patients with RA and HCs based on the KEGG database. Pathway enrichment analysis of M2c macrophages in patients with RA compared to HCs highlighting the fold change upregulation of pathways implicated in RA pathogenesis. A term gene graph of the associated enriched pathways highlighted the genes that were up- and downregulated. Fibro, fibroblasts; Endo, endothelial cells; NOD-like receptor, nucleotide-binding oligomerisation domain-like receptor; MAPK, mitogen-activated protein kinase; PI3K-Akt, phosphoinositide 3-kinase and protein kinase B.

Pathway enrichment analysis between RA and HC synovial macrophages was performed on individual macrophage clusters (**Figure 4F**). Pathway enrichment identified several pathways, previously implicated in RA pathogenesis which were enriched in RA compared to HC macrophages. Genes in the NOD-like receptor, MAPK and PI3K-Akt signalling pathways were highly enriched in RA M2c macrophages compared to HC, whilst efferocytosis-related genes, such as MerTK, were highly downregulated (**Figure 4F**). M1, M2a, M2b and M2c RA macrophages had enriched MAPK signalling pathway activity (**Supplementary Figure 3**). Additionally, PI3K-Akt signalling pathway was enriched in most but not all macrophage subsets in RA compared to HC (**Supplementary Figure 3**). The antigen presentation signalling pathway was significantly enriched in M2b, M2c and M2d macrophages of patients with RA, compared to their counterpart HC (**Figure 4F**; **Supplementary Figure 3**).

### Differential CD86 and MerTK expression by RA M2c macrophages under hypoxic conditions compared to HC.

A positive correlation between hypoxia in the joint of patients with RA, the degree of synovial inflammation and disease severity has previously been reported in RA (32). Hypoxia may be a possible primary driver of inflammatory processes in the arthritic joint, leading to increased infiltration of macrophages and T cells. Patient with RA-derived B cells are resistant to hypoxic conditions, whereby they produce more proinflammatory cytokines in the hypoxic environment compared to HC-derived PD-1+ B cells (52). Hence, polarisation of macrophages was paired at atmospheric 21% O_2_ and hypoxic 2% O_2_ for 48 hours, followed by RT-qPCR and flow cytometric analysis for characterisation of macrophages under hypoxia.

Differential gene expression profiles can be observed in HC between the different O_2_ environments (**Figure 5A**). The expression of most M2-related markers was downregulated in macrophages exposed to a hypoxic environment. Particularly, a significant downregulation of CD206 (M2a *p = 0.0404, M2c **p = 0.0014) and IL-10R (M1 *p = 0.0142 was observed across most hypoxic HC macrophage subtypes compared to paired HC macrophages polarised under atmospheric conditions (**Figure 5B**). Similar to HC, patient with RA macrophages display differences in their gene expression profile when exposed to a low O_2_ environment (**Figure 5C**). Downregulation of Arg1 was also observed in patient with RA macrophages under hypoxia compared to atmospheric conditions. In the case of RA-derived macrophages, a significant decrease in CD206 expression was observed in M2c (***p = 0.0002) macrophages under hypoxia, but not in M2a compared to the other macrophage subtypes (**Figure 5D**). No statistical differences in IL-10R expression were observed; however, CD163 was decreased in M2c (****p = 0.0001) RA-derived macrophages under hypoxia compared to atmospheric conditions (**Figure 5D**). A tendency toward higher VEGF expression by RA macrophages in hypoxia was also observed. This suggests that previous findings at atmospheric O_2_ need to be carefully interpolated similarly to what has been shown for B cells and fibroblasts in RA (52). Hence, these findings support our hypothesis that macrophage plasticity is dysregulated and supports the progression of disease, in the case of hypoxia, by downregulating anti-inflammatory mediators associated with the resolution of disease and wound healing.

**Figure 5:**
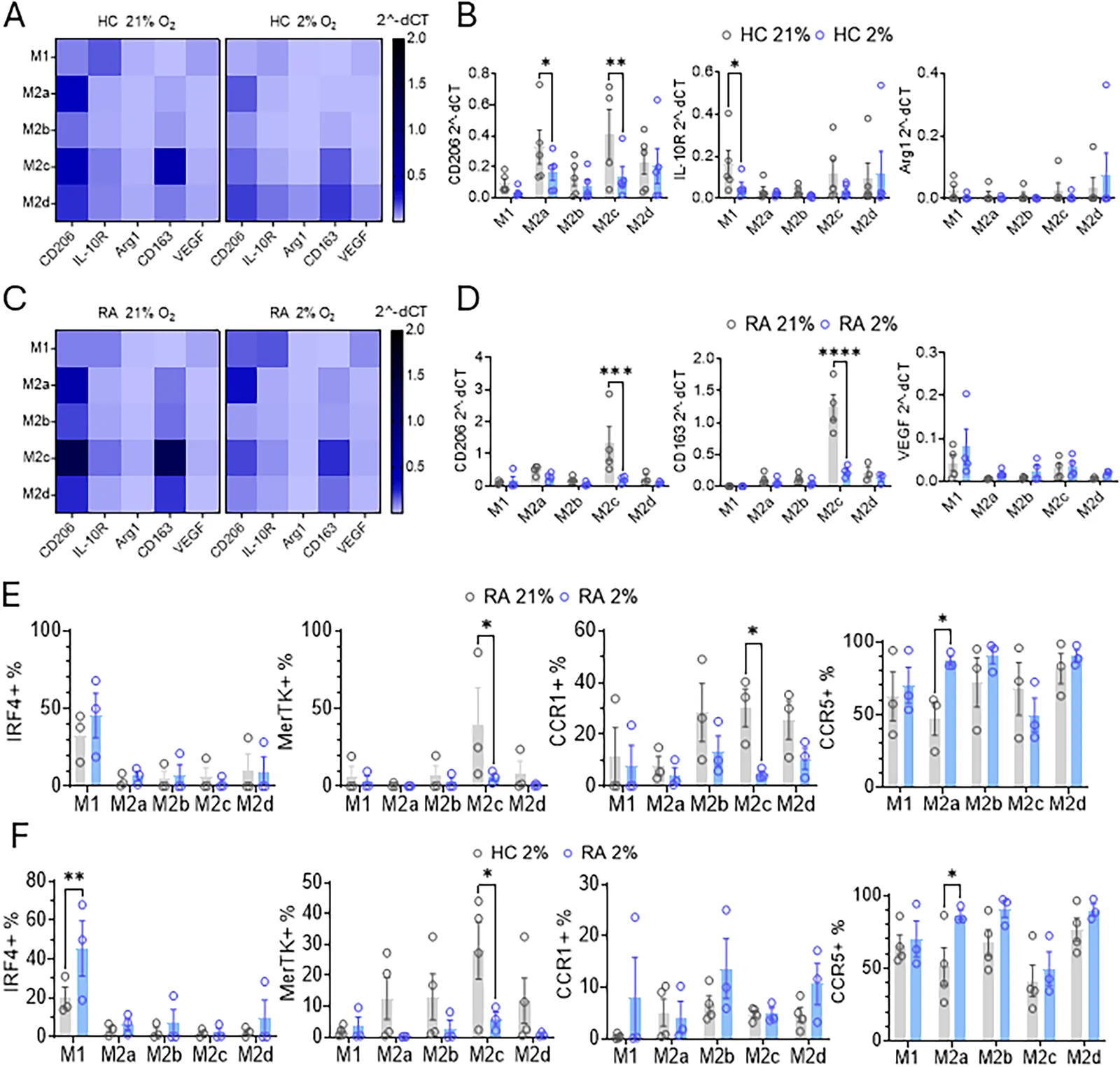
Hypoxia exacerbated the differences between RA and HC-derived macrophages. **(A)** Heatmaps displaying relative gene expression of macrophage markers (CD206, IL-10R, Arg1, CD163 and VEGF) by paired atmospheric and hypoxic RT-qPCR of HC macrophages (n=5). The scale represents 2^-dCT values whereby a low value signifies low expression relative to GAPDH expression. **(B)** Significant downregulation of M2 phenotype markers (CD206, IL-10R and Arg1) was observed in most HC macrophages polarised under hypoxia **(C)** Heatmaps of paired atmospheric and hypoxic RT-qPCR of patients with RA polarised macrophages (n=4). The scale represents 2^-dCT values in which a low value signifies low expression relative to GAPDH expression. **(D)** Differential gene expression of macrophage markers (CD206, CD163 and VEGF) by patient-derived macrophages in 2% O_2_ compared to 21% O_2_, assayed using RT-qPCR, showed a trend for downregulated expression in M2c macrophages exposed to hypoxia **(E)** Bar charts of paired flow cytometric analysis of intracellular (IRF4) and extracellular (MerTK, CCR1 and CCR5) marker expression in patients with RA macrophages (n=3) from hypoxic and atmospheric environments. **(F)** Bar charts comparing the effects of hypoxia on functional marker expression (IRF4, MerTK, CCR1 and CCR5) in patient with RA (n=3) and HC (n=4) macrophages, whereby hypoxia led an exacerbation of the differences previously observed between patient with RA and HC macrophages. Statistical analysis was performed by using 2-way ANOVA, *p < 0.05, **p < 0.01, ***p < 0.001, ****p < 0.0001.

Oxygen deprivation significantly downregulated MerTK expression by patient-derived hypoxic M2c macrophages compared to atmospheric M2c macrophages (*p = 0.0109) (**Figure 5E**). A similar decrease in expression of CCR1 was observed in M2c (*p = 0.0185) hypoxic macrophages. On the contrary, CCR5 was significantly upregulated by hypoxic M2a (*p = 0.0315) RA macrophages compared to atmospheric RA macrophages, whilst a marked decrease was observed by the M2c subset (**Figure 5E**). These differences are further exacerbated when compared to the HC-derived macrophages polarised under hypoxic conditions (**Figure 5F**). IRF4 expression was significantly higher in M1 RA-derived macrophages compared to HC (**p = 0.0099) (**Figure 5F**). No significant difference in CCR1 expression was observed between RA and HC macrophages under hypoxia. Importantly, a further decrease in MerTK expression was observed in M2c RA macrophages compared to hypoxic HC (*p = 0.014). CCR5 was further upregulated in RA M2a macrophages compared to HCs (*p = 0.0141) (**Figure 5F**). Hence, hypoxia aggravated the differences between patients with RA and HC macrophages, resulting in increased macrophage activation and migration to the site of inflammation. Consequently, these findings support our hypothesis that macrophage plasticity is dysregulated and supports the progression of disease, in the case of hypoxia, by downregulating anti-inflammatory mediators associated with the resolution of disease and wound healing and upregulating chemokines involved in chemotaxis to the site of inflammation.

### Differential OC phenotypes in RA and HC macrophage-derived OC.

Dysregulated macrophage plasticity may also lead to distinct OCs, in turn influencing disease pathogenesis. Therefore, the ability of blood-derived macrophage subsets to differentiate into OCs was tested. Following macrophage polarisation, the cells were subjected to differentiation towards OCs by stimulation with M-CSF and RANKL (**Figure 6A**). To confirm polarised macrophages differentiated into OCs, TRAP activity detection was performed on all macrophage subsets (**Figure 6B**; **Supplementary Figure 4A**). A significantly higher number of OCs formed from M1 (**p = 0.0098), M2a (*p = 0.0466), M2b (***p = 0.0002) and M2d (***p = 0.0003) RA-derived macrophages and almost none from M2c RA and HC macrophages (**Figure 6C**). The mean area of the differentiated OCs was higher in M2a (*p = 0.0328) RA macrophages compared to HC (**Figure 6C**). This suggests that they have a high propensity to differentiate into more pathogenic OCs as previous studies have shown that bigger, more nucleated OCs are more erosive in the joint (53).

**Figure 6:**
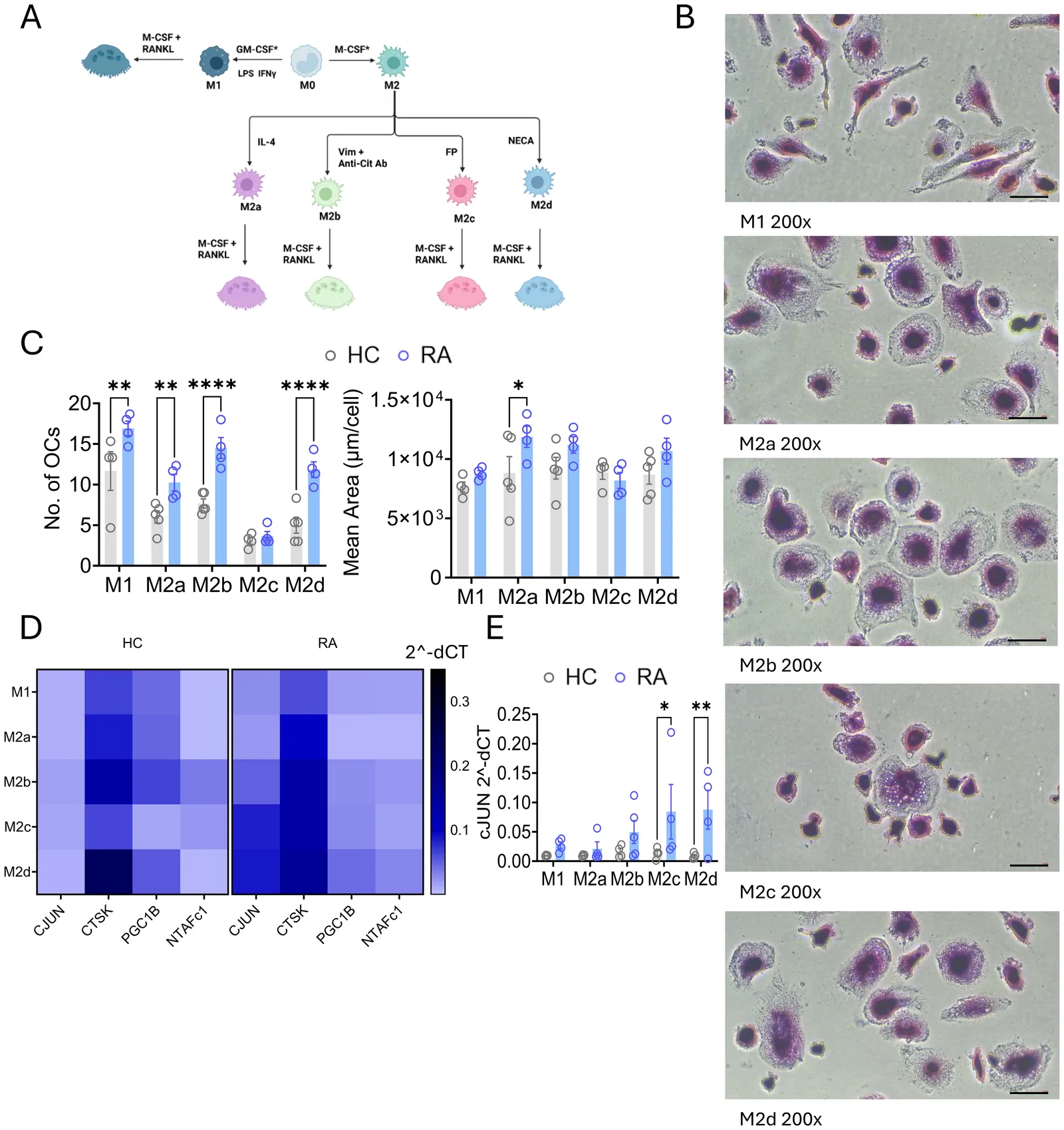
Differential phenotypic profile demonstrated in macrophage-derived osteoclasts in patients with RA vs HC. **(A)** Schematic of osteoclast differentiation protocol from polarised monocyte-derived macrophages. All macrophage subsets are subjected to stimulation to differentiate into osteoclasts (OCs) by the addition of macrophage colony-stimulating factor (M-CSF) and receptor activator of NF-κB ligand (RANKL) for 10 days. **(B)** TRAP-staining of macrophage-derived osteoclasts at 200x magnification. **(C)** Quantification of OC formation and the mean area (µm/cell) of the differentiated OCs in HC (n=5) and patients with RA (n=3). **(D)** Heatmaps of relative gene expression assayed via RT-qPCR and analysed by the 2^-dCT method of HC (n=5) and RA (n=4) osteoclasts derived from polarised macrophages. **(E)** Bar chart of 2^-dCT values of HC (n=4) and RA (n=5) OCs for cJUN expression, highlighting a significant increase in expression by M2c and M2d macrophages in patients with RA. Statistical analysis was performed by using 2-way ANOVA, *p < 0.05, **p < 0.01, ****p < 0.0001.

Differences were observed at the mRNA level of OCs via RT-qPCR using a panel of markers (cJUN, CTSK, PGC1β, NFATc1) associated with pathogenic aspects of OCs. **Figure 6D** highlights the expression patterns of RA and HC-derived OCs. cJUN, a transcription factor necessary for OC formation and activation, showed a significant increase in expression in M2c (*p = 0.0165) and M2d (**p = 0.0084) patients with RA macrophage-derived OCs compared to HC (**Figure 6E**). CTSK secreted by OCs plays a central role in bone remodelling processes in RA, with elevated levels correlating to radiological joint destruction (54). Patient-derived M2 macrophages differentiated into OCs showed a tendency for higher CTSK expression than M1 macrophages (**Figure 6D**; **Supplementary Figure 4B**). PGC1β, essential for OC function, showed a trend for lower expression in all RA macrophages compared to HC (**Figure 6D**; **Supplementary Figure 4B**). NFATc1, transcription regulator of OC differentiation, displayed a marked tendency for higher expression in M1-derived macrophages compared to HC (**Figure 6D**). Hence, distinct OC phenotypes were observed from the different macrophage subtypes.

## Discussion

The dogma of macrophage differentiation into proinflammatory M1 and anti-inflammatory M2 states has been challenged in recent years as a result of their plasticity and the discovery of macrophage subtypes, particularly within the M2 subset. This led to the hypothesis that the different macrophage subsets had alternate characteristic profiles in patients with RA compared to HC, and it is this plastic nature of macrophages, when dysregulated, that aids disease progression. Our study, for the first time, collectively characterised all known macrophage subtypes in RA.

M1 macrophages play a vital role in RA pathogenesis by amplifying and exacerbating the inflammatory site through their release of proinflammatory cytokines and chemokines (6). Meanwhile, the M2 macrophages are immunoregulatory with roles in wound healing and repair, in turn aiming to alleviate the symptoms of inflammation (13). However, the favourable manipulation of their plasticity towards an anti-inflammatory phenotype can be utilised to address the influence of proinflammatory mediators (55). This plasticity has been observed through the clear contrast between patients with RA and HC in their phenotypic, metabolic and functional profiles. Whilst RA has been associated with an imbalance of M1/M2 subset phenotype and cytokine activity, favouring the proinflammatory M1 macrophages (8–10), scRNAseq showed comparable distribution of synovial tissue macrophage subsets between HC and patients with RA. However, macrophage subtype-specific and global differences were observed in the transcriptomic profile of RA and HC synovial macrophages.

A typical proinflammatory macrophage profile was observed in M1 macrophages by their phenotype, metabolism and function. In line with previous studies, M1 macrophages demonstrated enhanced glycolytic capacity. They utilise glycolysis for their energy derivation, as a result of GLUT1 activating IL-1β (56). In line with previous findings by Jablonski et al., we showed that M1 macrophages differentiated in the presence of LPS have upregulated CD38 compared to M2 macrophages, in turn playing a part in their polarisation and proinflammatory function through their activation of the JAK-STAT pathway (57). M1 proinflammatory phenotype is reiterated through considerably higher secretion of proinflammatory cytokines in comparison to M2 macrophages. This accentuates their pathogenic function in RA progression through a positive feedback loop of proinflammatory mediator secretion and accumulation in the inflammatory site. Elevated VEGF expression in patients with RA compared to HC was seen in M1 macrophages. Previous studies have suggested the potential of M1 and M2d macrophage communication via IL-10, CCL3/CCR1 and VEGF signalling (27). In RA synovial M2d macrophages, a significant upregulation of CCR1 expression compared to HC was observed.

On the other hand, the M2 subtype macrophages, in line with current literature, had higher mitochondrial dependency than M1 macrophages in HC and RA, highlighting their dependency on OxPhos. M2 macrophages produce ATP by guiding pyruvate into the oxidative Krebs cycle, fuelled by mitochondrial FAO and glutamine metabolism (58). It has previously been described that HC M2a macrophages have the potential to undergo phenotypic and functional changes by repolarising towards an M2b-like phenotype in the presence of IgG4, which stimulates IL-10 and CCL1 secretion (19, 23). In this study, we highlighted the plastic nature of M2a macrophages via shifts in their energy derivation from OxPhos to an M1-like macrophage that prioritises glycolysis. This novel finding indicates that in an inflammatory microenvironment such as RA, M2a macrophages are highly plastic and can undertake both pro- and anti-inflammatory properties by expressing high levels of proinflammatory cytokines such as IL-1β, whilst also expressing higher quantities of CD206 and CD163 than in HC. This differs from current literature, which describes M2a macrophages as typically marked by CD206+CD163- cytokine expression and a highly activated arginase pathway (1, 59–61). M2b macrophages, on the other hand, showed a tendency for downregulated expression of CD206, IL-10R and Arg1 on an mRNA level in HC macrophages under hypoxic O_2_ conditions. Importantly, CD206 was further decreased in patient with RA M2b macrophages. As a result, M2b macrophages in the joints of patients with RA may contribute less effectively to tissue repair. This reinforces that hypoxia exacerbates the differences between patient with RA and HC macrophages. Consistent with our findings, M2b macrophages have also been associated with intermediate expression of proinflammatory cytokines such as IL-6, which allows them to regulate the extent of the immune response (62).

High expression of TNFα, as seen in M1 macrophages, are due to Fc receptors on the surface of macrophages directing their induction. Ultimately, this leads to the expression of RANKL, a receptor activator of the NF-κB ligand, which in turn leads to osteoclastogenesis, angiogenesis, bone and cartilage erosion and synovial hyperplasia (17). By characterising the ability of different macrophage subsets to differentiate into OCs, a potential mechanism for targeting macrophages and/or OCs to induce resolution of inflammation can be identified. To our knowledge, this is the first study to collectively characterise all known macrophage subsets differentiation efficiency into OCs and their phenotypic differences between patients with RA and HCs. Macrophage-derived OCs displayed an intense staining for TRAP, a histochemical marker of OCs (63). Patient with RA M2 macrophages formed significantly more and larger macrophage-derived OCs than in HCs, further exacerbating the disease course. This was seen in M2a, M2b and M2d macrophage subsets. M2c-derived OCs displayed resistance to adopting proinflammatory properties by resisting differentiation into OCs, highlighted by the high proportion of cell death after commencing differentiation. The significant upregulation of cJUN expression by M2c and M2d macrophages in patient-derived OCs suggests that patient macrophages are primed for OC formation. M1 macrophages have high osteoclastogenic function, as seen by M1-derived OCs which had a marked tendency for higher cJUN expression compared to HC, further highlighting their strong proinflammatory role in RA pathogenesis and association with OC formation. Dysregulated plasticity of macrophages may support the progression of RA through OC formation.

Since hypoxia simulates the inflammatory environment in the synovial tissue and joints of patients with RA, macrophages were polarised under hypoxic conditions. Interestingly, there was significant downregulation of MerTK expression in M2c RA macrophages under both atmospheric and hypoxic conditions. Previously, Alivernini et al. described a unique transcriptomic profile in STMs of HCs and patients with RA in remission that were mainly MerTK+CD206+ and associated with sustaining disease remission (6). These cells were CD163+ and consisted of two subpopulations, TREM2+ and FOLR2+LYVE1+, enriched in negative regulators of inflammation that reinstate tissue homeostasis (6). These subpopulations have complementary roles in regulating local immune responses and homeostasis, with the TREM2+ subset being a source of resolvins that induce repair processes in fibroblast-like synoviocytes (6). The FOLR2+LYVE1+ cluster upregulated genes involved in collagen turnover, coagulation factors and regulators of VEGF, thereby playing a role in tissue remodelling and homeostasis (6). In this study, M2c macrophages were marked by CD206 and CD163 and showed significantly higher expression of MerTK in both HC and patients with RA compared to the other subsets (40). CD163+ macrophages are known to infiltrate sites of inflammation and injury during the early stages of wound healing to induce cell clearance of apoptotic cells (23, 40). In addition to this, a reduced expression of proinflammatory mediators, such as CD38, was observed in M2c RA macrophages. Hence, the M2c macrophages in this study are consistent with the MerTK+ STMs described by Alivernini et al., whereby they displayed significantly higher expression of MerTK, TREM2 and FOLR2 in HC compared to patients with RA. Under homeostatic conditions, MerTK, TREM2 and FOLR2 control immune responses and maintain synovial homeostasis. However, in the hypoxic inflammatory microenvironment of patients with RA, MerTK is significantly downregulated in M2c macrophages and, in turn, may prevent remission of disease.

This study is limited to differentiating PBMCs instead of macrophages found directly in the joints of patients with RA, although the scRNAseq analysis was conducted on synovial cells. PBMCs polarised into macrophages may not be identical in behaviour to the macrophages found in the synovium of the inflamed joint. To overcome this, we compared the *in vitro* polarised macrophages to scRNAseq data from synovial macrophages of patients with RA and HC. Importantly, it was found that the transcriptomic profile of synovial macrophages matched the phenotypic characteristics observed in the blood-derived macrophages, thereby allowing us to confirm our results. M2c macrophages can be polarised *in vitro* by either utilisation of IL-10 or corticosteroids. Although both methods are widely used, the resulting M2c macrophages may show phenotypic as well as functional differences (64). In this study, we used corticosteroid-polarised M2c macrophages since RA patients are commonly exposed to corticosteroids during treatment. A limitation of this study is that the severe reduction in OC formation from corticosteroid-polarised M2c macrophages may be influenced by the macrophage polarisation method utilised in the study and should be assessed further against IL-10 polarised M2c macrophages. Our findings shed light on the distinct characteristics of M1, M2a, M2b, M2c and M2d macrophages in terms of their phenotypic, metabolic and functional profiles. Their dysregulated plasticity allows them to share characteristics of both M1 and M2 phenotypes, which may support the progression of RA. These alterations may impair tissue resolution, enhance osteoclastogenesis, and sustain chronic inflammation in the arthritic joint. Macrophage plasticity may be favourably manipulated as demonstrated by Li et al. (55); however, research is required. Subsequently, targeting/stabilising M2c macrophages and re-establishing homeostasis may be vital for tissue regeneration, inflammation resolution and the treatment of RA.

## Data Availability

The datasets presented in this study can be found in online repositories. The names of the repository/repositories and accession number(s) can be found below: https://www.ncbi.nlm.nih.gov/, GSE200815.

## References

[1] Kulakova K Lawal TR Mccarthy E Floudas A . The contribution of macrophage plasticity to inflammatory arthritis and their potential as therapeutic targets. Cells. (2024) 13:1586. doi: 10.3390/cells13181586 39329767 PMC11430612

[2] Fleischmann R Schiff M van der Heijde D Ramos-Remus C Spindler A Stanislav M . Baricitinib, methotrexate, or combination in patients with rheumatoid arthritis and no or limited prior disease-modifying antirheumatic drug treatment. Arthritis Rheumatol. (2017) 69:506–17. doi: 10.1002/art.39953 27723271 PMC5347954

[3] Söderlin MK Börjesson O Kautiainen H Skogh T Leirisalo-Repo M . Annual incidence of inflammatory joint diseases in a population based study in southern Sweden. Ann Rheum Dis. (2002) 61:911–5. doi: 10.1136/ard.61.10.911 PMC1753904 12228162

[4] Ambarus CA Noordenbos T de Hair MJ Tak PP Baeten DL . Intimal lining layer macrophages but not synovial sublining macrophages display an IL-10 polarized-like phenotype in chronic synovitis. Arthritis Res Ther. (2012) 14:R74. doi: 10.1186/ar3796 22494514 PMC3446447

[5] Diarra D Stolina M Polzer K Zwerina J Ominsky MS Dwyer D . Dickkopf-1 is a master regulator of joint remodeling. Nat Med. (2007) 13:156–63. doi: 10.1038/nm1538 17237793

[6] Alivernini S MacDonald L Elmesmari A Finlay S Tolusso B Gigante MR . Distinct synovial tissue macrophage subsets regulate inflammation and remission in rheumatoid arthritis. Nat Med. (2020) 26:1295–306. doi: 10.1038/s41591-020-0939-8 32601335

[7] Floudas A Smith CM Tynan O Neto N Krishna V Wade SM . Distinct stromal and immune cell interactions shape the pathogenesis of rheumatoid and psoriatic arthritis. Ann Rheum Dis. (2022) 81:1224–42. doi: 10.1136/annrheumdis-2021-221761 35701153

[8] Lin Y Wang Z Liu S Liu J Zhang Z Ouyang Y . Roles of extracellular vesicles on macrophages in inflammatory bone diseases. Mol Cell Biochem. (2024) 479:1401–14. doi: 10.1007/s11010-023-04809-w 37436653

[9] Qadri M Jay GD Zhang LX Schmidt TA Totonchy J Elsaid KA . Proteoglycan-4 is an essential regulator of synovial macrophage polarization and inflammatory macrophage joint infiltration. Arthritis Res Ther. (2021) 23:241. doi: 10.1186/s13075-021-02621-9 34521469 PMC8439011

[10] Liu Y Hao R Lv J Yuan J Wang X Xu C . Targeted knockdown of PGAM5 in synovial macrophages efficiently alleviates osteoarthritis. Bone Res. (2024) 12:1–12. doi: 10.1038/s41413-024-00318-8 38433252 PMC10909856

[11] Fukui S Iwamoto N Takatani A Igawa T Shimizu T Umeda M . M1 and M2 monocytes in rheumatoid arthritis: A contribution of imbalance of M1/M2 monocytes to osteoclastogenesis. Front Immunol. (2017) 8:1958. doi: 10.3389/fimmu.2017.01958 PMC5766997 29375576

[12] Paoletti A Rohmer J Ly B Pascaud J Rivière E Seror R . Monocyte/macrophage abnormalities specific to rheumatoid arthritis are linked to miR-155 and are differentially modulated by different TNF inhibitors. J Immunol. (2019) 203:1766–75. doi: 10.4049/jimmunol.1900386 31484730 PMC6755128

[13] Iwasaki M Kamiya K Murata S Maekawa T Komine M Ohtsuki M . Involvement of M1/M2 macrophages in the pathomechanisms of intralymphatic histiocytosis associated with rheumatoid arthritis. J Dermatol. (2019) 46:e42–e43. doi: 10.1111/1346-8138.14509 29905385

[14] van Hamburg JP Asmawidjaja PS Davelaar N Mus AM Colin EM Hazes JM . Th17 cells, but not Th1 cells, from patients with early rheumatoid arthritis are potent inducers of matrix metalloproteinases and proinflammatory cytokines upon synovial fibroblast interaction, including autocrine interleukin-17A production. Arthritis Rheum. (2011) 63:73–83. doi: 10.1002/art.30093 20954258

[15] Kovács OT Tóth E Ozohanics O Soltész-Katona E Marton N Buzás EI . Proteomic changes of osteoclast differentiation in rheumatoid and psoriatic arthritis reveal functional differences. Front Immunol. (2022) 13. doi: 10.3389/fimmu.2022.892970 PMC9289121 35860269

[16] Clavel C Nogueira L Laurent L Iobagiu C Vincent C Sebbag M . Induction of macrophage secretion of tumor necrosis factor α through Fcγ receptor IIa engagement by rheumatoid arthritis–specific autoantibodies to citrullinated proteins complexed with fibrinogen. Arthritis Rheum. (2008) 58:678–88. doi: 10.1002/art.23284 18311806

[17] Mathsson L Lampa J Mullazehi M Rönnelid J . Immune complexes from rheumatoid arthritis synovial fluid induce FcγRIIa dependent and rheumatoid factor correlated production of tumour necrosis factor-α by peripheral blood mononuclear cells. Arthritis Res Ther. (2006) 8:R64. doi: 10.1186/ar1926 16569263 PMC1526644

[18] Vlk AM Prantner D Shirey KA Perkins DJ Buzza MS Thumbigere-Math V . M2a macrophages facilitate resolution of chemically-induced colitis in TLR4-SNP mice. mBio. (2023) 14:e01208-23. doi: 10.1128/mbio.01208-23 37768050 PMC10653841

[19] Bianchini R Roth-Walter F Ohradanova-Repic A Flicker S Hufnagl K Fischer MB . IgG4 drives M2a macrophages to a regulatory M2b-like phenotype: potential implication in immune tolerance. Allergy. (2019) 74:483–94. doi: 10.1111/all.13635 PMC6492166 30338531

[20] Rőszer T . Understanding the mysterious M2 macrophage through activation markers and effector mechanisms. Mediators Inflammation. (2015) 2015:816460. doi: 10.1155/2015/816460 PMC4452191 26089604

[21] Ross EA Devitt A Johnson JR . Macrophages: The good, the bad, and the gluttony. Front Immunol. (2021) 12:708186. doi: 10.3389/fimmu.2021.708186 34456917 PMC8397413

[22] Yang J Zhang X Chen J Heng BC Jiang Y Hu X . Macrophages promote cartilage regeneration in a time- and phenotype-dependent manner. J Cell Physiol. (2022) 237:2258–70. doi: 10.1002/jcp.30694 35147979

[23] Lurier EB Dalton D Dampier W Raman P Nassiri S Ferraro NM . Transcriptome analysis of IL-10-stimulated (M2c) macrophages by next-generation sequencing. Immunobiology. (2017) 222:847–56. doi: 10.1016/j.imbio.2017.02.006 28318799 PMC5719494

[24] Junior Lai Y-S Nguyen HT Salmanida FP Chang K-T . MERTK+/hi M2c macrophages induced by baicalin alleviate non-alcoholic fatty liver disease. Int J Mol Sci. (2021) 22:10604. doi: 10.3390/ijms221910604 34638941 PMC8508959

[25] Fabriek BO Dijkstra CD van den Berg TK . The macrophage scavenger receptor CD163. Immunobiology. (2005) 210:153–60. doi: 10.1016/j.imbio.2005.05.010 16164022

[26] Ferrante CJ Pinhal-Enfield G Elson G Cronstein BN Hasko G Outram S . The adenosine-dependent angiogenic switch of macrophages to an M2-like phenotype is independent of interleukin-4 receptor alpha (IL-4Rα) signaling. Inflammation. (2013) 36:921–31. doi: 10.1007/s10753-013-9621-3 23504259 PMC3710311

[27] Lai W Xian C Chen M Luo D Zheng J Zhao S . Single-cell and bulk transcriptomics reveals M2d macrophages as a potential therapeutic strategy for mucosal healing in ulcerative colitis. Int Immunopharmacol. (2023) 121:110509. doi: 10.1016/j.intimp.2023.110509 37369160

[28] Culemann S Grüneboom A Nicolás-Ávila JA Weidner D Lämmle KF Rothe T . Locally renewing resident synovial macrophages provide a protective barrier for the joint. Nature. (2019) 572:670–5. doi: 10.1038/s41586-019-1471-1 31391580 PMC6805223

[29] Feng D Huang WY Niu XL Hao S Zhang LN Hu YJ . Significance of macrophage subtypes in the peripheral blood of children with systemic juvenile idiopathic arthritis. Rheumatol Ther. (2021) 8:1859–70. doi: 10.1007/s40744-021-00385-x 34693468 PMC8572293

[30] Argüello RJ Combes AJ Char R Gigan JP Baaziz AI Bousiquot E . SCENITH: A flow cytometry based method to functionally profile energy metabolism with single cell resolution. Cell Metab. (2020) 32:1063–1075.e7. doi: 10.1016/j.cmet.2020.11.007 PMC8407169 33264598

[31] Roederer M Nozzi JL Nason MC . SPICE: Exploration and analysis of post-cytometric complex multivariate datasets. Cytometry A. (2011) 79A:167–74. doi: 10.1002/cyto.a.21015 21265010 PMC3072288

[32] Ng CT Biniecka M Kennedy A McCormick J Fitzgerald O Bresnihan B . Synovial tissue hypoxia and inflammation *in vivo*. Ann Rheum Dis. (2010) 69:1389–95. doi: 10.1136/ard.2009.119776 20439288 PMC2946116

[33] Hanlon MM Smith CM Canavan M Neto NGB Song Q Lewis MJ . Loss of synovial tissue macrophage homeostasis precedes rheumatoid arthritis clinical onset. Sci Adv. (2024) 10:eadj1252. doi: 10.1126/sciadv.adj1252 39321281 PMC11423874

[34] McGinnis CS Murrow LM Gartner ZJ . DoubletFinder: Doublet detection in single-cell RNA sequencing data using artificial nearest neighbors. Cell Syst. (2019) 8:329–337.e4. doi: 10.1016/j.cels.2019.03.003 30954475 PMC6853612

[35] Hafemeister C Satija R . Normalization and variance stabilization of single-cell RNA-seq data using regularized negative binomial regression. Genome Biol. (2019) 20:296. doi: 10.1186/s13059-019-1874-1 31870423 PMC6927181

[36] Korsunsky I Millard N Fan J Slowikowski K Zhang F Wei K . Fast, sensitive and accurate integration of single-cell data with Harmony. Nat Methods. (2019) 16:1289–96. doi: 10.1038/s41592-019-0619-0 31740819 PMC6884693

[37] Ulgen E Ozisik O Sezerman OU . pathfindR: An R package for comprehensive identification of enriched pathways in omics data through active subnetworks. Front Genet. (2019) 10. doi: 10.3389/fgene.2019.00858 31608109 PMC6773876

[38] Cutolo M Campitiello R Gotelli E Soldano S . The role of M1/M2 macrophage polarization in rheumatoid arthritis synovitis. Front Immunol. (2022) 13. doi: 10.3389/fimmu.2022.867260 35663975 PMC9161083

[39] Cutolo M Soldano S Gotelli E Montagna P Campitiello R Paolino S . CTLA4-Ig treatment induces M1–M2 shift in cultured monocyte-derived macrophages from healthy subjects and rheumatoid arthritis patients. Arthritis Res Ther. (2021) 23:306. doi: 10.1186/s13075-021-02691-9 34952630 PMC8709961

[40] Zizzo G Hilliard BA Monestier M Cohen PL . Efficient clearance of early apoptotic cells by human macrophages requires “M2c” polarization and MerTK induction. J Immunol Baltim Md 1950. (2012) 189:3508–20. doi: 10.4049/jimmunol.1200662 22942426 PMC3465703

[41] Guo D Lin C Lu Y Guan H Qi W Zhang H . FABP4 secreted by M1-polarized macrophages promotes synovitis and angiogenesis to exacerbate rheumatoid arthritis. Bone Res. (2022) 10:1–15. doi: 10.1038/s41413-022-00211-2 35729106 PMC9213409

[42] Paoletti A Ly B Cailleau C Gao F de Ponfilly-Sotier MP Pascaud J . Liposomal AntagomiR-155-5p restores anti-inflammatory macrophages and improves arthritis in preclinical models of rheumatoid arthritis. Arthritis Rheumatol. (2024) 76:18–31. doi: 10.1002/art.42665 37527031

[43] Srirangan S Choy EH . The role of interleukin 6 in the pathophysiology of rheumatoid arthritis. Ther Adv Musculoskelet Dis. (2010) 2:247–56. doi: 10.1177/1759720x10378372 22870451 PMC3383508

[44] Liu J Cao S Kim S Chung EY Homma Y Guan X . Interleukin-12: An update on its immunological activities, signaling and regulation of gene expression. Curr Immunol Rev. (2005) 1:119–37. doi: 10.2174/1573395054065115 21037949 PMC2965603

[45] Muench DE Sun Z Sharma A Tang C Crampton JS Lao C . A pathogenic Th17/CD38+ macrophage feedback loop drives inflammatory arthritis through TNF-α. J Immunol Baltim Md 1950. (2022) 208:1315–28. doi: 10.4049/jimmunol.2101025 35197330

[46] Marzaioli V Canavan M Floudas A Flynn K Mullan R Veale DJ . CD209/CD14+ dendritic cells characterization in rheumatoid and psoriatic arthritis patients: Activation, synovial infiltration, and therapeutic targeting. Front Immunol. (2022) 12. doi: 10.3389/fimmu.2021.722349 35095831 PMC8789658

[47] Chan HC Wang SC Lin CH Lin YZ Li RN Yen JH . A novel CD209 polymorphism is associated with rheumatoid arthritis patients in Taiwan. J Clin Lab Anal. (2021) 35:e23751. doi: 10.1002/jcla.23751 33792986 PMC8128313

[48] Mamun A Liu F . Role of IRF4-mediated inflammation: Implication in neurodegenerative diseases. Neurol Neurother Open Access J. (2017) 2:107. doi: 10.23880/nnoaj-16000107 PMC11521387 39473489

[49] Shi Z Rifa'i M Lee YH Shiku H Isobe K Suzuki H . Importance of CD80/CD86–CD28 interactions in the recognition of target cells by CD8+CD122+ regulatory T cells. Immunology. (2008) 124:121–8. doi: 10.1111/j.1365-2567.2007.02747 PMC2434386 18205792

[50] Liu MF Kohsaka H Sakurai H Azuma M Okumura K Saito I . The presence of costimulatory molecules CD86 and CD28 in rheumatoid arthritis synovium. Arthritis Rheum. (1996) 39:110–4. doi: 10.1002/art.1780390115 8546719

[51] Liu MF Kohsaka H Sakurai H Azuma M Okumura K Saito I . MerTK signaling in macrophages promotes the synthesis of inflammation resolution mediators by suppressing CaMKII activity. Sci Signal. (2018) 11:eaar3721. doi: 10.1126/scisignal.aar3721 30254055 PMC6171110

[52] Cai B Kasikara C Doran AC Ramakrishnan R Birge RB Tabas I . Pathogenic, glycolytic PD-1^+^ B cells accumulate in the hypoxic RA joint. JCI Insight. (2020) 5. doi: 10.1172/jci.insight.139032 33148884 PMC7710281

[53] Takito J Nakamura M . Heterogeneity and actin cytoskeleton in osteoclast and macrophage multinucleation. Int J Mol Sci. (2020) 21:6629. doi: 10.3390/ijms21186629 32927783 PMC7554939

[54] Floudas A Neto N Marzaioli V Murray K Moran B Monaghan MG . Serum cathepsin K levels of patients with longstanding rheumatoid arthritis: Correlation with radiological destruction. Arthritis Res Ther. (2005) 7:R65–70. doi: 10.1186/ar1461 PMC1064888 15642144

[55] Li H Feng Y Zheng X Jia M Mei Z Wang Y . M2-type exosomes nanoparticles for rheumatoid arthritis therapy *via* macrophage re-polarization. J Controlled Release. (2022) 341:16–30. doi: 10.1016/j.jconrel.2021.11.019 34793917

[56] Luo P Du M Sun Q Zhao T He H . IL-38 suppresses macrophage M1 polarization to ameliorate synovial inflammation in the TMJ via GLUT-1 inhibition. Int Immunopharmacol. (2023) 122:110619. doi: 10.1016/j.intimp.2023.110619 37463548

[57] Jablonski KA Amici SA Webb LM Ruiz-Rosado JD Popovich PG Partida-Sanchez S . Novel markers to delineate murine M1 and M2 macrophages. PloS One. (2015) 10:e0145342. doi: 10.1371/journal.pone.0145342 26699615 PMC4689374

[58] Xiao H Guo Y Li B Li X Wang Y Han S . M2-like tumor-associated macrophage-targeted codelivery of STAT6 inhibitor and IKKβ siRNA induces M2-to-M1 repolarization for cancer immunotherapy with low immune side effects. ACS Cent Sci. (2020) 6:1208–22. doi: 10.1021/acscentsci.9b01235 32724855 PMC7379385

[59] Li D Ma H Shu Q Wang T Li L Huang P . Arsenite inhibits M2a polarization of macrophages through downregulation of peroxisome proliferator-activated receptor gamma. Toxicol Appl Pharmacol. (2022) 450:116142. doi: 10.1016/j.taap.2022.116142 35777529

[60] Zizzo G Hilliard BA Monestier M Cohen PL . Efficient clearance of early apoptotic cells by human macrophages requires M2c polarization and MerTK induction. J Immunol Baltim Md 1950. (2012) 189:3508–20. doi: 10.4049/jimmunol.1200662 22942426 PMC3465703

[61] Wang Q Sudan K Schmoeckel E Kost BP Kuhn C Vattai A . CCL22-polarized TAMs to M2a macrophages in cervical cancer *in vitro* model. Cells. (2022) 11:2027. doi: 10.3390/cells11132027 35805111 PMC9265611

[62] Huang S Yue Y Feng K Huang X Li H Hou J . Conditioned medium from M2b macrophages modulates the proliferation, migration, and apoptosis of pulmonary artery smooth muscle cells by deregulating the PI3K/Akt/FoxO3a pathway. PeerJ. (2020) 8:e9110. doi: 10.7717/peerj.9110 32411539 PMC7207208

[63] Hayman AR . Tartrate-resistant acid phosphatase (TRAP) and the osteoclast/immune cell dichotomy. Autoimmunity. (2008) 41:218–23. doi: 10.1080/08916930701694667 18365835

[64] Huang X Li Y Fu M Xin H-B . Polarizing macrophages *in vitro*. Methods Mol Biol. (2018) 119–126. doi: 10.1007/978-1-4939-7837-3_12 29761394 PMC8875934

